# Dynamic Rewiring of the *Drosophila* Retinal Determination Network Switches Its Function from Selector to Differentiation

**DOI:** 10.1371/journal.pgen.1003731

**Published:** 2013-08-29

**Authors:** Mardelle Atkins, Yuwei Jiang, Leticia Sansores-Garcia, Barbara Jusiak, Georg Halder, Graeme Mardon

**Affiliations:** 1 Program in Developmental Biology, Baylor College of Medicine, Houston, Texas, United States of America; 2 VIB Center for the Biology of Disease, KU Leuven Center for Human Genetics, University of Leuven, Leuven Belgium; 3 Department of Molecular and Human Genetics, Baylor College of Medicine, Houston, Texas, United States of America; 4 Department of Developmental Biology, University of Texas Southwestern Medical Center at Dallas, Dallas, Texas, United States of America; 5 Department of Pathology, Baylor College of Medicine, Houston, Texas, United States of America; 6 Department of Neuroscience, Baylor College of Medicine, Houston, Texas, United States of America; 7 Department of Ophthalmology, Baylor College of Medicine, Houston, Texas, United States of America; New York University, United States of America

## Abstract

Organ development is directed by selector gene networks. Eye development in the fruit fly *Drosophila melanogaster* is driven by the highly conserved selector gene network referred to as the “retinal determination gene network,” composed of approximately 20 factors, whose core comprises *twin of eyeless* (*toy*), *eyeless* (*ey*), *sine oculis* (*so*), *dachshund* (*dac*), and *eyes absent* (*eya*). These genes encode transcriptional regulators that are each necessary for normal eye development, and sufficient to direct ectopic eye development when misexpressed. While it is well documented that the downstream genes *so*, *eya*, and *dac* are necessary not only during early growth and determination stages but also during the differentiation phase of retinal development, it remains unknown how the retinal determination gene network terminates its functions in determination and begins to promote differentiation. Here, we identify a switch in the regulation of *ey* by the downstream retinal determination genes, which is essential for the transition from determination to differentiation. We found that central to the transition is a switch from positive regulation of *ey* transcription to negative regulation and that both types of regulation require *so*. Our results suggest a model in which the retinal determination gene network is rewired to end the growth and determination stage of eye development and trigger terminal differentiation. We conclude that changes in the regulatory relationships among members of the retinal determination gene network are a driving force for key transitions in retinal development.

## Author Summary

Animals develop by using different combinations of simple instructions. The highly conserved retinal determination (RD) network is an ancient set of instructions that evolved when multicellular animals first developed primitive eyes. Evidence suggests that this network is re-used throughout evolution to direct the development of organs that communicate with the brain, providing information about our internal and external world. This includes our eyes, ears, kidneys, and pancreas. An upstream member of the network named *eyeless* must be activated early to initiate eye development. Eyeless then activates the expression of downstream genes that maintain *eyeless* expression and define the eye field. Here, we show that *eyeless* must also be turned off for final steps of eye development. We investigated the mechanism by which *eyeless* is turned off and we find that feedback regulation by the downstream RD genes changes to repress Eyeless expression during late stages of development. This study shows that tight regulation of *eyeless* is important for normal development and provides a mechanism for its repression.

## Introduction

During organogenesis, cells undergo progressive cell fate restriction coupled with a loss of pluripotency. This process is hallmarked by the stages of specification, proliferation, and differentiation [1]. The transitions between each of these states mark major changes in developmental competence and plasticity during tissue and organ development.

The adult fly eye develops from a larval structure called the eye imaginal disc [2], [3]. Following specification and growth during early larval development, the retinal field begins to differentiate during the third larval stage, or instar [4]. *Drosophila* eye differentiation occurs progressively, proceeding from the posterior to the anterior margins of the disc; its progress is marked by a morphologically and molecularly detectable event called the morphogenetic furrow [5]–[7]. Anterior to the morphogenetic furrow, cells are determined and proliferating, while posterior to it cells exit the cell cycle and differentiate. Within the morphogenetic furrow, cells transition from proliferation to differentiation. Thus, the developing *Drosophila* eye is an ideal system to study how cells regulate the transition from pluripotency to terminal differentiation.

**Figure 1:**
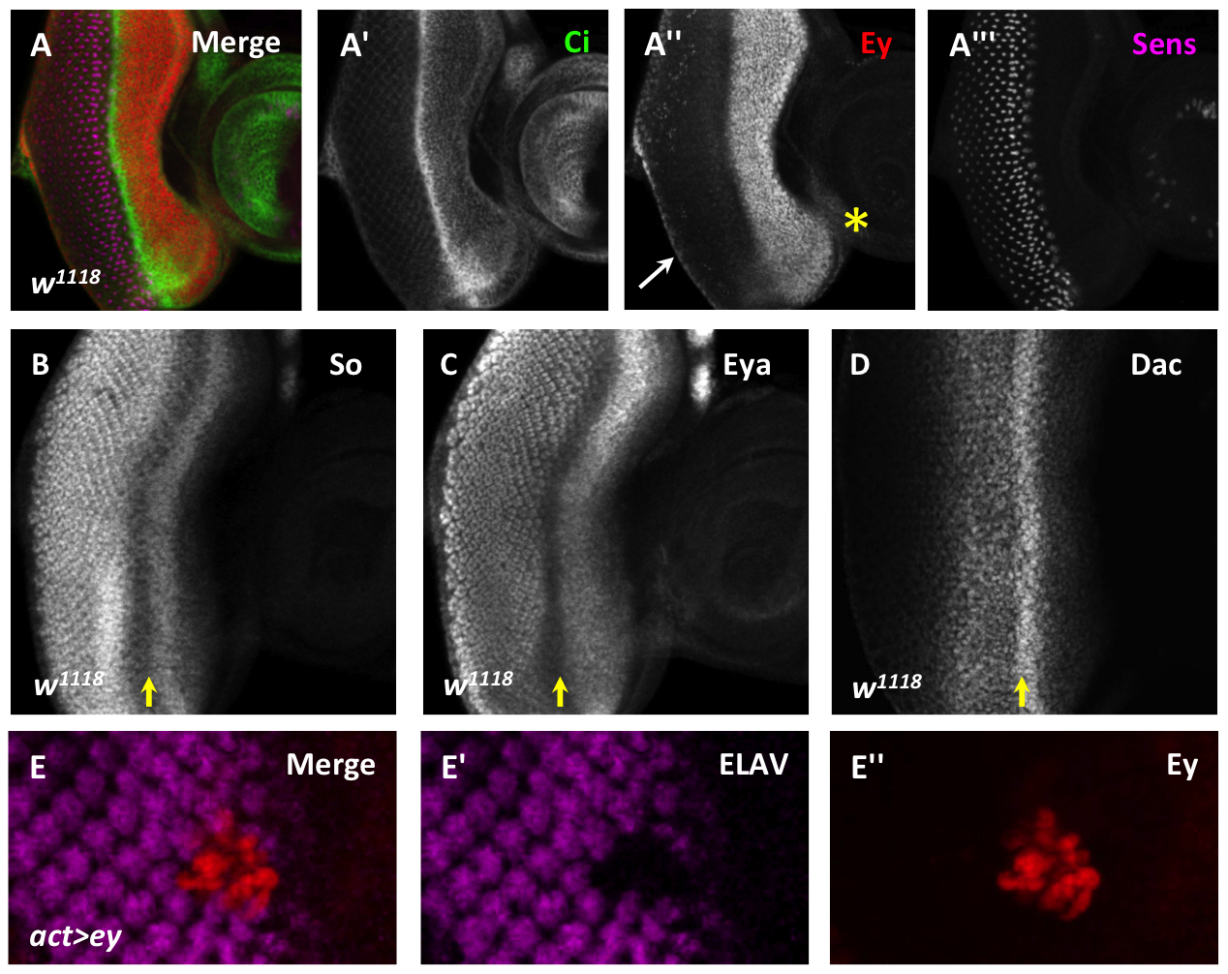
Ey repression at the morphogenetic furrow is necessary for differentiation. (A) Cubitus interruptus (Ci), Eyeless (Ey), and Senseless (Sens) expression in a *w^1118^* third instar eye-antennal imaginal disc. (A′) Strong Ci accumulation marks the morphogenetic furrow. (A″) Ey expression; white arrow marks cuboidal margin cells, yellow asterisk marks ventral head capsule. (A′″) Sens expression shows R8 differentiation. (B–D) Yellow arrow marks the morphogenetic furrow: (B) Sine oculis (So) expression, (C) Eyes absent (Eya) expression, (D) Dachshund (Dac) expression. (E) Overexpression of Ey posterior to the morphogenetic furrow using *Flipout-Gal4* inhibits photoreceptor differentiation. (E′) ELAV from panel E showing differentiation. (E″) Eyeless expression from panel E.

Selector genes direct the development of many organs from their primordia [8]. The development of the eye imaginal disc into the adult eye is directed by a conserved network of transcriptional regulators called the retinal determination (RD) gene network. The core members of this network, *twin of eyeless* (*toy*), *eyeless* (*ey*), *sine oculis* (*so*), *eyes absent* (*eya*), and *dachshund* (*dac*), are each necessary for normal eye development and are sufficient to drive ectopic eye development in other imaginal discs [9]–[17]. During normal development, Toy activates *ey* expression in the first instar [17]. Initially, Ey is expressed throughout the disc and activates the expression of *eya*, *so*, and *dac* [18]–[21]. Once established, So maintains its own expression, as well as that of *dac* and *ey* [19], [22]. Such positive feedback mechanisms within the network are well characterized [17]–[19], [23]–[25]. The downstream RD network members Eya, So, and Dac are expressed and necessary in cells posterior to the morphogenetic furrow (Figure 1B–D) [9]–[13], [22], [26]. In contrast, at the morphogenetic furrow, *ey* expression is sharply down-regulated (Figure 1A), but how the positive feedback loops are terminated remains unknown [14], [18].

In the region just anterior to the morphogenetic furrow where Dac, Eya, So, and Ey overlap, these proteins cooperate to initiate the expression of low levels of the proneural gene *atonal* (*ato*), which is required for the onset of photoreceptor differentiation [27]–[29]. However, without further amplification and refinement by Notch signaling in the morphogenetic furrow, the low level of Ato expression induced in this region of the eye is not sufficient to induce photoreceptor differentiation, and Ey expression persists [30]–[32]. Thus, while RD gene activity is required to initially activate one of the most upstream genes required for the onset of differentiation, this is not sufficient to fully trigger differentiation.

In this work, we show that maintaining expression of *ey* posterior to the morphogenetic furrow blocks photoreceptor differentiation. In addition, we identify a key regulatory switch in the RD gene network required for the repression of *ey*. Specifically, So directly regulates *ey* anterior to the furrow to promote high levels of expression, and via the same enhancer binding site blocks high levels of *ey* expression posterior to the furrow. Our results support a model that *ey* expression posterior to the furrow is regulated indirectly by *eya* and *dac* expression, and is triggered by signaling events in the morphogenetic furrow. These results suggest a model in which rewiring of the RD gene network is a key driving force during retinal organogenesis.

## Results

### Ey repression is necessary for the onset of differentiation

During the third instar, Eyeless (Ey) is strongly expressed anterior to the morphogenetic furrow. However, its expression sharply decreases at the morphogenetic furrow, and is detected only weakly in the differentiating eye field (Figure 1A). In contrast, the downstream RD gene network members are expressed not only in undifferentiated cells anterior to the morphogenetic furrow, but also in differentiating cells posterior to the morphogenetic furrow (Figure 1B–D). To determine if reducing Ey expression at the morphogenetic furrow is important for normal eye development, we overexpressed Ey posterior to the furrow using two methods. First, using the Flipout-Gal4 system we generated clones of cells that maintained Ey expression beyond the passage of the furrow [12], [33]. This caused cells to fail to differentiate, as assayed by expression of the pan-neuronal marker ELAV (Figure 1E). Second, we reactivated Ey expression in cells posterior to the furrow using the *GMR-Gal4* and *lz-Gal4* drivers [34], [35]. *GMR-Gal4* eventually drives expression in all cells posterior to the furrow, while *lz-Gal4* drives expression in cells that generate the future photoreceptors R1, 6, and 7 as well as in the cone and pigment cell precursors. ELAV expression is not affected in these genotypes, suggesting that Ey is not sufficient to block differentiation once differentiation has begun (Figure S1A–C). However, adult eyes of *lz-Gal4; UAS-ey* show defects in ommatidial shape and pigment when compared to wild-type (Figure S1D,E). Sections through *lz-Gal4; UAS-ey* eyes showed that photoreceptors survive, but that rhabdomere morphogenesis and ommatidial rotation are abnormal, suggesting that terminal differentiation events are disrupted by ectopic Ey expression (Figure S1F,G). From these results we conclude that down-regulation of Ey expression is necessary for normal photoreceptor differentiation.

### So maintains Ey in determined cells and represses Ey in differentiating cells

**Figure 2:**
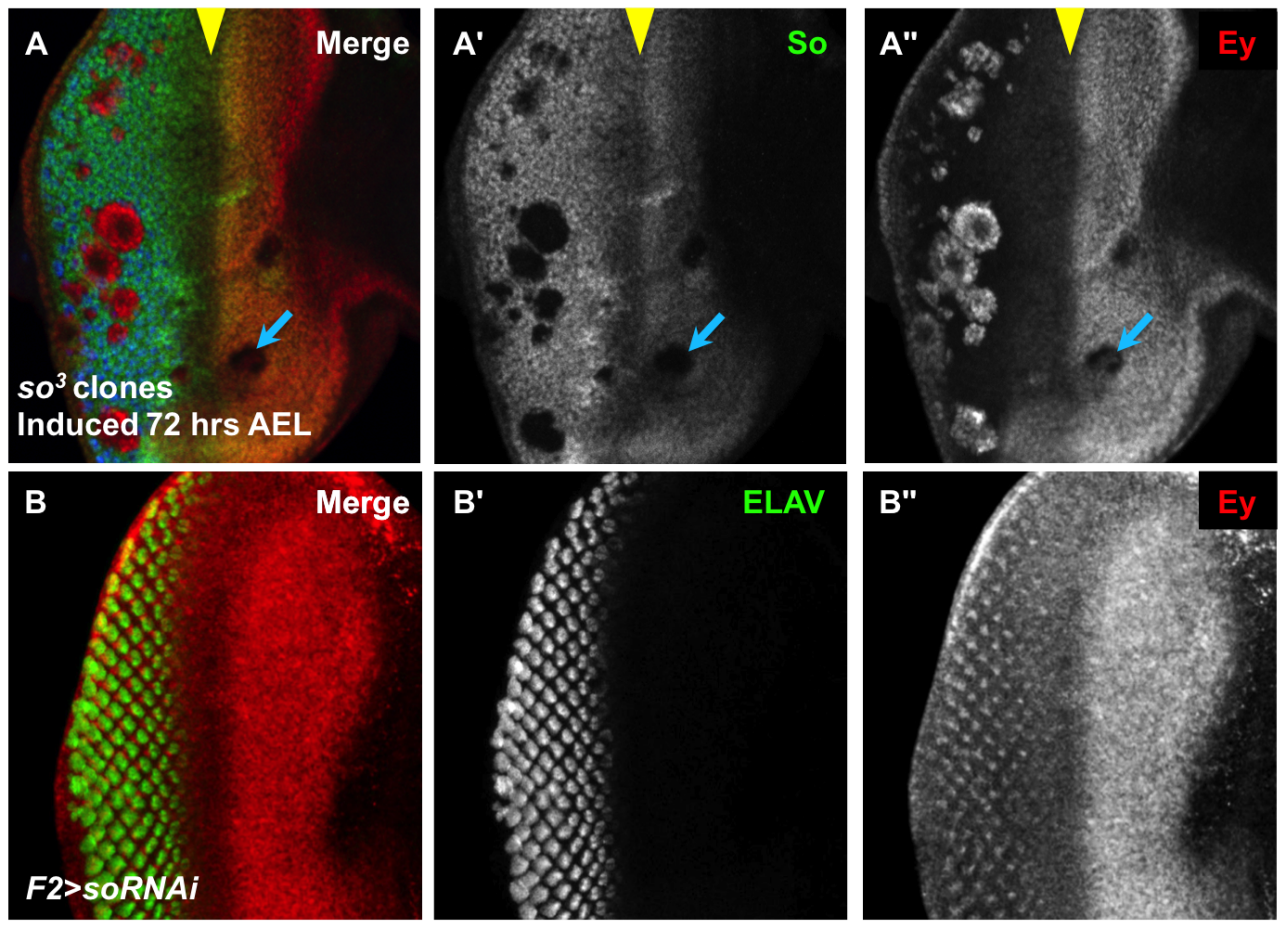
So regulates Ey expression anterior and posterior to the morphogenetic furrow. (A) *so^3^* null clones, induced by *hs-flp* 72 hrs AEL showing So (green), Ey (red), and ELAV expression (blue); the yellow arrowhead marks the morphogenetic furrow, and the blue arrow indicates an anterior clone. (A′) Grayscale image of So expression, green in A; loss of So expression marks the clones. (A″) Grayscale image of Ey, red in A. (B) *F2-Gal4* drives expression of *soRNAi* (VDRC transformant KK108128). (B′) Grayscale image of ELAV expression, green in B, marks differentiating photoreceptors. (B″) Grayscale image of Ey expression, red in B. Ey derepression posterior to the furrow matches previously described pattern of *F2-Gal4* expression.

To identify how the change in Ey expression is regulated, we undertook a candidate gene approach based on the literature. Previous studies of the RD gene network member Sine oculis (So) indicate that So activates *ey* expression during the third instar; however, *so* loss-of-function clones posterior to the morphogenetic furrow contained Ey expression, suggesting either that So is also required to suppress Ey expression or alternatively that these cells are trapped in an earlier developmental state [18], [22], [36]. This apparent paradox in the literature led us to examine Ey expression in *so^3^* null clones in different positions of the eye disc during the third instar. In *so^3^* clones anterior to the morphogenetic furrow, Ey expression was reduced, supportive of the model that So positively regulates *ey* expression anterior to the furrow (Figure 2A, arrow) [22]. Posterior to the morphogenetic furrow, we observed strong Ey expression in *so^3^* clones (Figure 2A) [36]. We conclude that So promotes Ey expression anterior to the furrow and suppresses Ey expression posterior to the furrow.

We investigated the non-uniform appearance of Ey expression in posterior *so^3^* clones, and observed that it is due to the morphology of the clones (Figure 2A). Specifically, orthogonal sections through clones displayed a spherical shape, with Ey expression being restricted to the *so* mutant tissue (Figure S2A,B). To determine if these cells lie in the interior of the clones that express low levels of Ey or no Ey, we co-labeled *so^3^* clones for both Ey and Lamin, a marker of the nuclear membrane. We observed spaces within the clones that lack nuclei, and these spaces lack Ey (Figure S2C). Therefore, we conclude that Ey is robustly expressed cell autonomously in all *so* mutant cells posterior to the furrow. Our clonal analyses suggest that So cell autonomously promotes Ey expression anterior to the morphogenetic furrow, and suppresses Ey expression posterior to the morphogenetic furrow.

The presence of *ey* transcript or protein in *so* loss-of-function clones posterior to the morphogenetic furrow has been interpreted previously as a secondary consequence of failed furrow progression and/or differentiation [26], [36]. However, it may be that *so* expression is required posterior to the morphogenetic furrow to negatively regulate Ey. To distinguish between these models, we let Ey undergo normal regulation anterior to and within the morphogenetic furrow and then knocked down *so* expression specifically in differentiating cells posterior to the morphogenetic furrow. The *F2-Gal4* driver, generated by our group with a characterized enhancer of the *sens* gene [37], initiates expression in the intermediate clusters within the furrow, posterior to Ey negative regulation, and is ultimately refined to drive expression most strongly in the R8 photoreceptor (Figure S2D,F). This driver permits analysis of the role of *so* in Ey regulation specifically in differentiating cells. Additionally, changes in expression are easily detectable because normal cells surround the knockdown cells. In *F2-Gal4>UAS-so-RNAi* discs, we observed Ey expression posterior to the morphogenetic furrow in an R8-like pattern (Figure 2B). Knockdown of So in *F2-Gal4>UAS-so-RNAi* discs is supported by So staining (Figure S2E) and results in a mildly disorganized adult eye (Figure S2G). Based on these results, we conclude that *so* is required to suppress Ey expression posterior to the morphogenetic furrow and that such suppression is required for normal eye development.

### A single So binding site is required for *ey* maintenance and suppression

**Figure 3:**
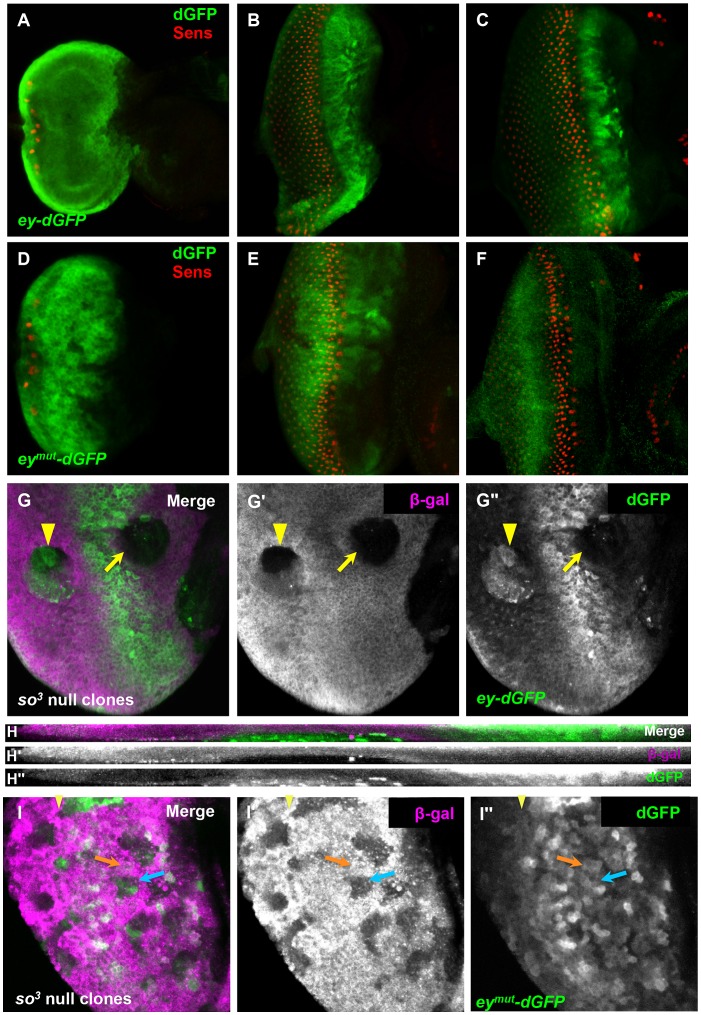
So regulates *ey* expression through a binding site in an *ey* eye enhancer. (A–F) reporter expression in third instar discs; columns of Sens positive cells were counted to compare furrow progression at different times. (A–C) expression of *ey-dGFP* (green) and Sens (red) in early (one column of photoreceptors) (A), mid (12 columns of photoreceptors) (B) and late (20 columns of photoreceptors) (C) third instar eye imaginal discs; individual channels shown in Figure S3. (D–F) expression of *ey^mut^-dGFP* (green) and Sens (red) in third instar eye imaginal discs; individual channels shown in Figure S3. (D) one column of photoreceptors, (E) 11 columns of photoreceptors, (F) 18 columns of photoreceptors. (G) *ey-dGFP* expression in *so^3^* null clone anterior (yellow arrow) and posterior (yellow arrowhead) to the morphogenetic furrow. (G′) Grayscale image of β-Galactosidase expression, magenta in G; loss of β-Galactosidase marks the clone (G″) Grayscale image of *ey-dGFP* expression, green in G (H–H″) Maximum projection of orthogonal sections through the posterior clone indicated by a yellow arrowhead in G–G″. (I) *ey^mut^-dGFP* (green) expression in *so^3^* null clone marked by loss of β-Galactosidase expression (magenta) in a disc aged between panels D and E. The yellow arrowhead marks the furrow; the orange arrow indicates non-clone tissue, blue arrow indicates anterior clone; similar expression detected in and out of clone (I′) Grayscale image of β-Galactosidase expression, magenta in I. (I″) Grayscale image of *ey-dGFP* expression, green in I.

So is a homeodomain transcription factor, leading us to ask if So suppresses *ey* expression at the transcriptional level. To test this, we required a reporter that recapitulates *ey* regulation anterior and posterior to the morphogenetic furrow. Published *ey* enhancer reporters [22], [38], unlike Ey expression, persist posterior to the morphogenetic furrow, possibly due to perdurance of beta-galactosidase. We therefore constructed a new destabilized GFP (dGFP) reporter. To compare wild-type and mutant constructs while avoiding position effects, we utilized a vector that could integrate only at specific sites in our analysis [39]–[41]. We cloned a previously characterized full-length eye enhancer from the *ey* locus into this new dGFP vector, “*ey-dGFP”* [38], [39]. We detected robust expression with *ey-dGFP* throughout larval development (Figure 3A–C, Figure S3A–C). Similar to *ey* expression, *ey-dGFP* is expressed throughout the eye disc in first instar (not shown) and is maintained throughout the eye disc until furrow initiation (Figure 3A). During the third instar *ey-dGFP* is maintained anterior to the morphogenetic furrow and suppressed at the morphogenetic furrow, similar to Ey expression (Figure 3B). This expression pattern is maintained throughout the third instar (Figure 3C). Therefore, this enhancer recapitulates the Ey expression pattern in the eye field.

To determine if *ey-dGFP* can be regulated by So, we generated *so^3^* clones and assayed reporter expression in clones anterior and posterior to the furrow. As with Ey, *ey-dGFP* reporter expression was reduced in anterior *so^3^* clones, while it was induced in posterior clones (Figure 3G–H). Based on these results, we conclude that So regulates *ey* expression at the transcriptional level both anterior and posterior to the morphogenetic furrow.

To determine if So can regulate the expression of *ey-dGFP* directly, we mutated a previously well-characterized So binding site in the *ey* enhancer to generate *ey^mut^-dGFP* [22]. From early development through initiation of the morphogenetic furrow *ey^mut^-dGFP* is indistinguishable from *ey-dGFP*, consistent with published data that early *ey* expression is independent of So (Figure 3D, Figure S3D) [18]. However, during furrow progression, the expression pattern of *ey^mut^-dGFP* is dynamic. The expression of *ey^mut^-dGFP* anterior to the morphogenetic furrow is initially strong but weakens throughout the third instar, and eventually becomes barely detectable (Figure 3E,F, Figure S3E,F). This may indicate that additional positive regulators of *ey* are initially expressed in this domain, consistent with findings that Tsh promotes Ey expression in the same region [42], [43]. This is also consistent with our observation that Ey expression is diminished but not lost in the anterior *so^3^* clones we observed (Figure 2A). By the time the furrow has progressed 7–8 columns, *ey^mut^-dGFP* expression is detected posterior to the onset of Sens expression in the furrow. By 14 columns of photoreceptor recruitment, *ey^mut^-dGFP* is expressed in most cells posterior to the morphogenetic furrow (Figure 3E, Figure S3E shows a disc at 11 columns). Posterior expression is detected weakly even in very late discs where anterior expression is lost (Figure 3F, Figure S3F shows a disc of 18 columns) suggesting that the So binding site is required posterior to the furrow to suppress activation of *ey* by another activator. We conclude that a So binding site is required to suppress expression of the *ey* enhancer reporter posterior to the furrow and to maintain reporter expression anterior to the furrow.

To determine if So can regulate *ey^mut^-dGFP* expression, we examined *ey^mut^-dGFP* expression in *so^3^* clones. If mutation of the binding site is sufficient to make the reporter unresponsive to regulation by So, then we should not observe changes in the reporter expression pattern when we compare tissue within versus outside of clones. We chose to assay a time point early in furrow progression when the reporter is still expressed anterior to the furrow and is beginning to express posterior to it. We observed areas of identical reporter brightness both inside and outside of the clones, leading us to conclude that mutation of the binding site makes the reporter unresponsive to regulation by So (Figure 3I). Together with the fact that this binding site has been demonstrated to be bound by So in vitro [22], our analyses of *ey-dGFP* and *ey^mut^-dGFP* lead us to conclude that So directly regulates the expression of Ey both anterior and posterior to the morphogenetic furrow through the same binding site.

### The So cofactor Eya is necessary for Ey repression

We next wanted to investigate the mechanism by which So represses Ey posterior to the furrow. Sine oculis interacts with multiple cofactors that affect its function as a transcriptional regulator, including the transcriptional coactivator Eyes absent (Eya) and the TLE family corepressor Groucho (Gro) [25], [36], [44]–[46]. As both cofactors are expressed in the eye disc, we set out to determine which of them, if either, cooperates with So to regulate Ey. We performed loss-of-function analyses for each cofactor and assayed the effects on Ey expression in clones. Our primary candidate was Gro, which cooperates with So in the repression of targets in the eye [25], [46]. Surprisingly, null loss-of-function clones of *gro* had no effect on Ey expression anterior or posterior to the morphogenetic furrow (Figure S4A). We conclude that Gro is not necessary for the normal regulation of Ey expression during the third instar, and unlikely to cooperate with So in this process.

**Figure 4:**
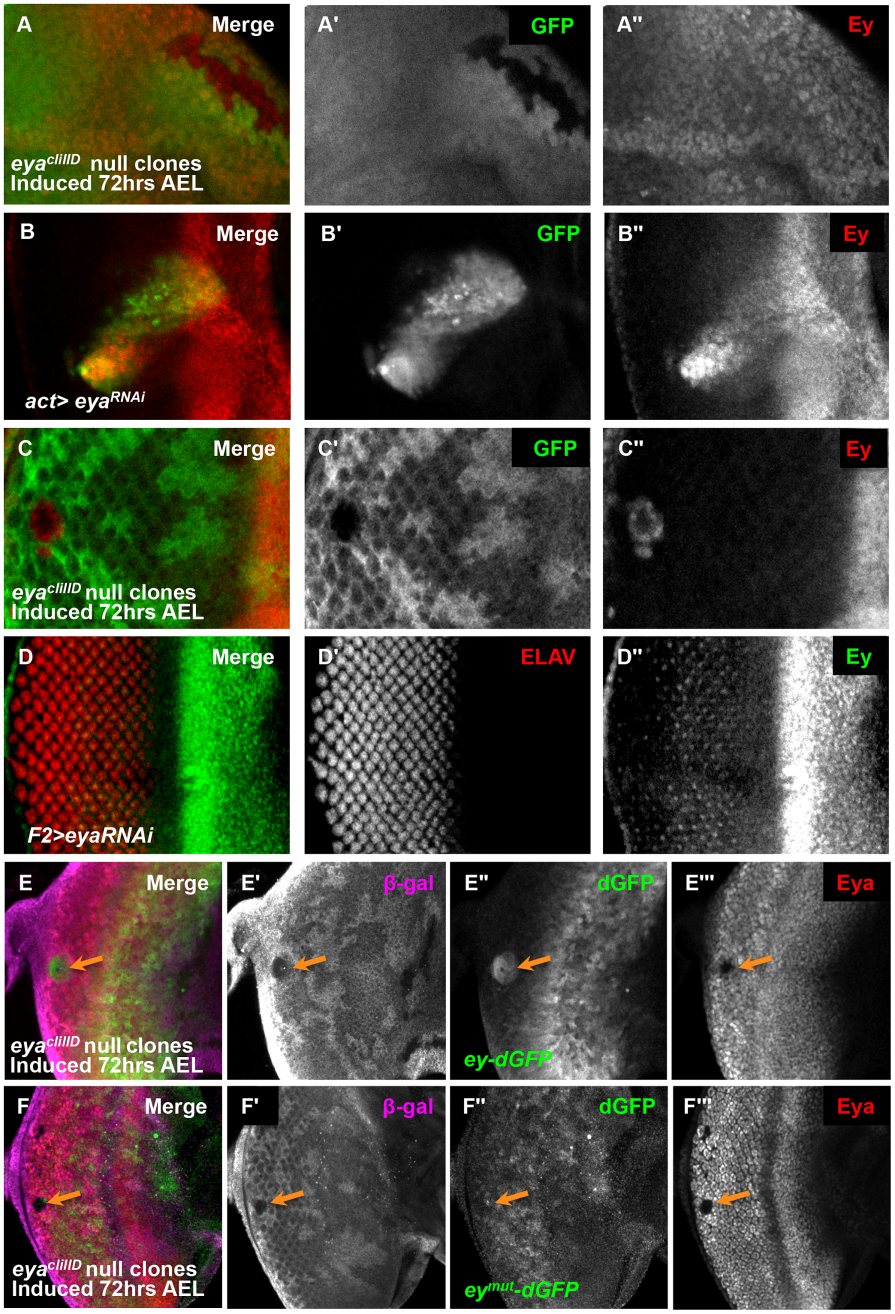
Eya is necessary for *ey* repression posterior to the morphogenetic furrow. (A) *eya^cliIID^* null clones anterior to the morphogenetic furrow, induced by *hs-flp* 72 hrs AEL showing GFP and Ey expression (A′) Grayscale image of GFP expression, shown as green in A; loss of GFP expression marks the clones. (A″) Grayscale image of Ey channel alone, red in A. (B) *Flipout-Gal4* drives expression of *eyaRNAi* (VDRC transformant KK108071). Merge of GFP and Ey expression shown. (B′) Grayscale image of GFP expression, shown as green in B; GFP expression marks the clones. (B″) Grayscale image of Ey channel alone, red in B. (C) *eya^cliIID^* null clones posterior to the morphogenetic furrow, induced by *hs-flp* 72 hours after egg lay (AEL) showing GFP and Ey expression (C′) Grayscale image of GFP expression, shown as green in C; loss of GFP expression marks the clones. (C″) Grayscale image of Ey channel alone, red in C. (D) *F2-Gal4* drives expression of *eyaRNAi* (VDRC transformant KK108071). Merge of ELAV and Ey expression shown. (D′) Grayscale image of ELAV expression, shown as green in D, marks differentiating photoreceptors. (D″) Grayscale image of Ey expression, shown as red in D. (E) *ey-dGFP* (green) expression in *eya^cliIID^* null clone posterior to the morphogenetic furrow (indicated by orange arrow) marked by loss of β-Galactosidase expression (magenta) and Eya (red). (E′) Grayscale image of β-Galactosidase expression in E. (E″) Grayscale image of *ey-dGFP* expression in E as revealed by anti-GFP. (E′″) Grayscale image of Eya expression in E. (F) *ey^mut^-dGFP* (green) expression in *eya^cliIID^* null clone posterior to the morphogenetic furrow (indicated by orange arrow) marked by loss of β-Galactosidase expression (magenta) and Eya (red). (F′) Grayscale image of β-Galactosidase expression in F. (F″) Grayscale image of *ey-dGFP* expression in F as revealed by anti-GFP. (F′″) Grayscale image of Eya expression in F.

We next wanted to determine if Eya cooperates with So to regulate *ey*. Previous studies found that So and Eya physically interact to promote the activation of target genes [28], [36], [45], [47], [48]. Based on these studies, we predicted that *eya* would be necessary for the maintenance of Ey expression by So anterior to the morphogenetic furrow. To test this, we generated *eya* null clones and examined Ey expression. We observed, surprisingly, that Ey expression was normal in *eya* anterior clones (Figure 4A). As these clones were small and rare, we also used RNAi to knockdown *eya* expression using the Flipout-Gal4 technique. Even in large knock-down clones we observed that Ey expression was normal in clones anterior to the morphogenetic furrow (Figure 4B). These results indicate that Eya is not required to maintain Ey expression anterior to the furrow. Posterior to the furrow, both null and RNAi knockdown clones of *eya* expressed Ey strongly (Figure 4B,C). We also observed similar morphology changes in *eya* clones as in *so* clones posterior to the furrow (compare Figure 4C to Figure 2D). Based on these results, we conclude that *eya* expression is required for Ey suppression posterior to the furrow.

Eya is necessary for furrow progression and differentiation; therefore, failure of morphogenetic furrow progression through *eya* clones could result in the maintenance of Ey in these clones [26], [36], [49]–[51]. To test if Ey expression in posterior *eya* clones is an indirect effect of failed furrow progression, we used the *F2-Gal4* driver to knock down *eya* expression specifically posterior to the furrow. We observed Ey expression in *eya* knockdown cells (Figure 4D). Staining for Eya indicates that the RNAi effectively knocks down *eya* expression (Figure S5A). Adults of *F2-Gal4>eyaRNAi* have disorganized eyes (Figure S5B). We conclude that Eya is required for Ey suppression posterior to the furrow.

To determine if *eya* is required for Ey suppression at the transcriptional level and dependent upon the So binding site, we examined *ey-dGFP* and *ey^mut^-dGFP* expression in posterior *eya* clones. In clones posterior to the furrow, *ey-dGFP* was expressed, similar to *so* clone phenotypes, suggesting that *eya* is required for the negative regulation of *ey* at the transcriptional level (Figure 4E). In contrast to *ey-dGFP*, the expression of *ey^mut^-dGFP* is not induced in posterior *eya* clones, suggesting that it no longer requires *eya* for its regulation (Figure 4F). From these results we conclude that Eya regulation of *ey* requires the So binding site.

### High levels of *eya* and *so* are sufficient to repress endogenous Ey

**Figure 5:**
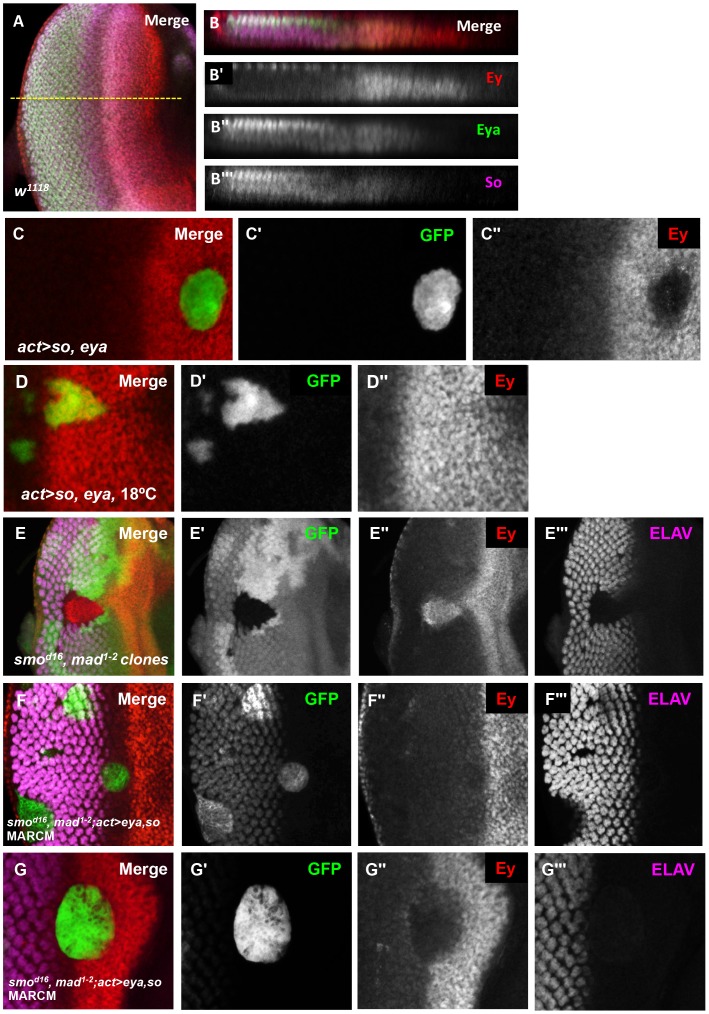
Eya and So can cooperate to negatively regulate Ey expression in vivo. (A) Expression patterns of Ey, Eya, and So in a *w^1118^* third instar eye imaginal disc. The yellow dashed line indicates the approximate location of the orthogonal section in B–B′″. (B) Orthogonal section of A. (B′) Grayscale image of Ey expression, red in B, apical Ey expression is detected in the peripodium, sections excluded in normal Z projection. (B″) Grayscale image of Eya expression, green in B. (B′″) Grayscale image of So expression, magenta in B. (C–G) Expression of *UAS-GFP* marks the clone(C, D, F, G); lack of GFP marks the clone in E. Crosses were raised at 25°C, except D, raised at 18°C. (C) *UAS-so* and *UAS-eya* were co-overexpressed anterior to the furrow. (C′) Grayscale image of GFP expression in C. (C″) Grayscale image of Ey expression in C. (D) *Flipout-Gal4* was used to co-express *UAS-so*, *UAS-eya*, and *UAS-GFP*. (D′) Grayscale image of GFP expression, green in D. (D″) Grayscale image of Ey expression, red in D. (E) Double loss of function clones for *smo^d16^* null allele and *mad^1–2^* hypomorphic allele were generated by inducing *hs-flp* expression at 48 hrs AEL. (E′) Grayscale image of GFP expression, green in E. (E″) Grayscale image of Ey expression, red in E. (E′″) Grayscale image of ELAV expression, magenta in E, shows differentiating photoreceptors. (F) MARCM clones that are mutant for *smo^d16^* and *mad^1–2^* while overexpressing *so* and *eya*. (F′) Grayscale image of GFP expression in F; the ELAV-like pattern is due to non-specific antibody interaction. (F″) Grayscale image of Ey expression in F. (F′″) Grayscale image of ELAV expression in F shows differentiating photoreceptors. (G–G′″) Same as F showing a clone extending anterior to the furrow.

Eya and So each overlap Ey expression just anterior to the morphogenetic furrow, but do not negatively regulate Ey expression in this zone. Therefore, we re-examined the expression of Eya and So in the eye imaginal disc to determine if their expression patterns could suggest how Eya and So could be required to suppress *ey* expression posterior to the furrow. Quantification of Eya and So expression in orthogonal sections revealed that expression of both factors is increased posterior to the morphogenetic furrow (Figure 5A,B). To test if the increased level is sufficient to repress Ey, we overexpressed both *eya* and *so* within the Ey domain using the Flipout-Gal4 strategy. Co-misexpression of *eya* and *so* was sufficient to repress Ey expression to background levels within the eye field, while ectopic Ey expression was detected in clones in other discs (Figure 5C, and data not shown). These data suggest that, within the developing retinal field, increased *so* and *eya* expression is sufficient to repress Ey expression anterior to the morphogenetic furrow. When we utilized the temperature sensitivity of the Gal4-UAS system to overexpress *eya+so* at 18°C, which results in lower expression of *eya+so* than at 25°C, they failed to repress Ey expression in the eye field, but were still sufficient to ectopically activate Ey expression in the antennal disc (Figure 5D, Figure S6A, white arrow).

The levels of So and Eya expression increase posterior to the morphogenetic furrow in response to activation of the Hedgehog (Hh) and Decapentaplegic (Dpp) signaling pathways [26]. Next, we asked if upregulation of Eya and So is sufficient to suppress *ey* even without the signaling pathways normally required for morphogenetic furrow movement. To test this, we made use of the MARCM system. We overexpressed *eya* and *so* simultaneously in *smo^3^*, *mad^1–2^* double mutant clones, which cannot respond to either Hh or Dpp signaling. Clones doubly mutant for these two signaling effectors are known to lack furrow progression: they do not activate Notch signaling, they lack differentiation, and they retain Ey expression [5], [26], [49], [52]–[54] (Figure 5E). We observed that Ey is strongly repressed in clones anterior to the morphogenetic furrow, and is not expressed in clones posterior to the morphogenetic furrow (Figure 5F,G). Therefore, high levels of *eya* and *so* are sufficient to repress Ey in the absence of normal morphogenetic furrow signaling. Together, these data suggest that the increased levels of Eya and So induced by signals in the morphogenetic furrow are important for Ey repression.

### Excess So can block *ey* transcription

**Figure 6:**
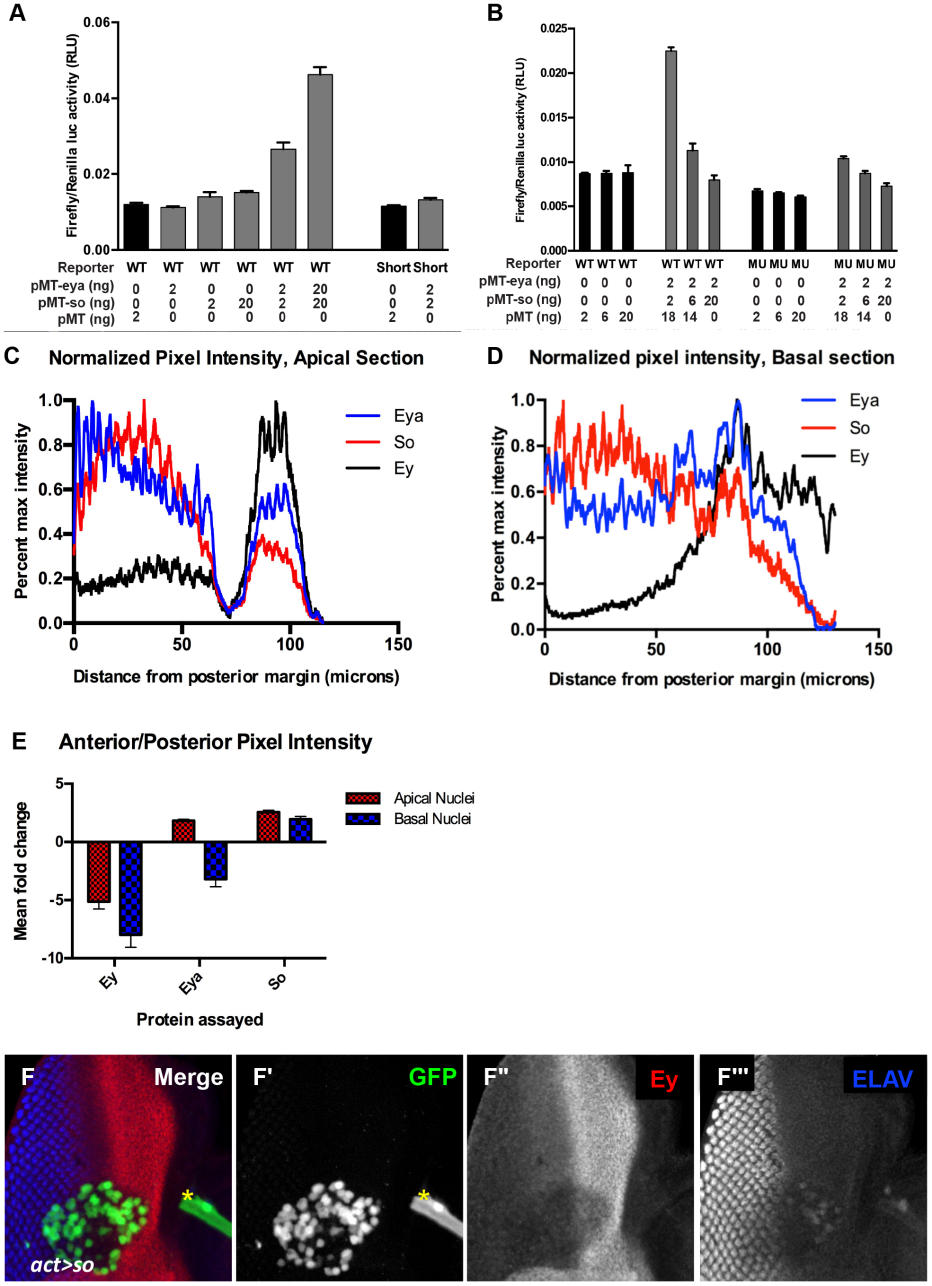
Excess So is sufficient to block enhancer activation in vitro. (A,B) Luciferase assay performed in transiently transfected S2 cells, reported as a ratio between Firefly and Renilla luciferase levels. Reporter constructs as indicated: WT = *ey-Luc*, Short = *ey^short^-Luc* that deletes the So binding site, MU = *ey^mut^-Luc*. Controls are graphed in black; manipulations are in gray. For each condition the nanograms transfected of pMT-eya, pMT-so, or pMT empty vector are indicated. (C,D) Normalized pixel intensity plots for fluorescent immunohistochemistry to assay Eya (blue), So (red), and Ey (black) expression in a *w^1118^* disc (see Materials and Methods). (C) Staining intensity in apical nuclei. (D) Staining intensity in basal nuclei. (E) Mean fold change for each channel was calculated (n = 5), and plotted. Error bars indicate S.E.M. (F) *Flipout-Gal4* driving *UAS-GFP* and *UAS-So* expression showing GFP, Ey, and ELAV. Yellow asterisk denotes a piece of trachea that is not part of the disc. (F′–F″) individual channels from panel F. (F′) GFP marks the clone, (F″) Ey, (F′″) ELAV shows differentiating photoreceptors.

To gain a better understanding of how Eya and So cooperate to regulate *ey* expression, we tested the response of the *ey* enhancer in vitro to So and/or Eya. In *Drosophila* S2 cells, when the *ey* enhancer is used to drive luciferase expression (*ey-luc*), reporter expression was induced by co-expression of So with Eya, but not by either factor alone (Figure 6A, “WT”). This suggests that the *ey* enhancer can be activated by Eya and So, and is consistent with previously published results that they cooperate to activate targets [45], [47]. Mutation or deletion of the So binding site (Mut or Short, respectively) within the reporter strongly reduced its induction by Eya/So (Figure 6A,B). This suggests that the activation of the construct in our assay depends primarily upon the So binding site.

Our in vivo results indicate that high levels of Eya and So expression can repress Ey expression. However, even a 10 fold increase of both transfected plasmids did not repress; rather, the reporter was activated more strongly (Figure 6A). To generate additional hypotheses we re-examined the in vivo expression of Ey, So, and Eya. We quantified pixel intensity values for Eya, So, and Ey in orthogonal sections (as in Figure 5B) across multiple imaginal discs (n = 5) as a proxy to examine expression levels across the third instar disc. Values were normalized and plotted for each protein to generate a line graph that visually depicts staining intensity across the section (as shown in Figure 6C,D). We observed that So undergoes a greater average positive fold change (Posterior Max/Anterior max) than Eya in both apical and basal sections (Figure 6E). While this analysis is only semi-quantitative, it was highly reproducible, and could indicate that So is in excess to Eya in posterior cells. At a minimum it suggests that their relative levels of expression are different in anterior and posterior cells. To test the simple model that excess So can prevent *ey* expression, we increased the ratio of transfected *so* plasmid to *eya* plasmid in our in vitro system. In response, we observed a dramatic decrease of reporter expression (Figure 6B), leading us to conclude that excess So suppresses activation of *ey-dGFP* by the Eya/So complex in vitro. To test this model in vivo we overexpressed So anterior to the morphogenetic furrow. We observed that in some clones Ey expression was mildly repressed by overexpression of So (Figure 6F). Based on our in vivo and in vitro observations, we conclude that excess So expression can be sufficient to suppress *ey* expression.

### Dac contributes to Ey repression within the morphogenetic furrow

**Figure 7:**
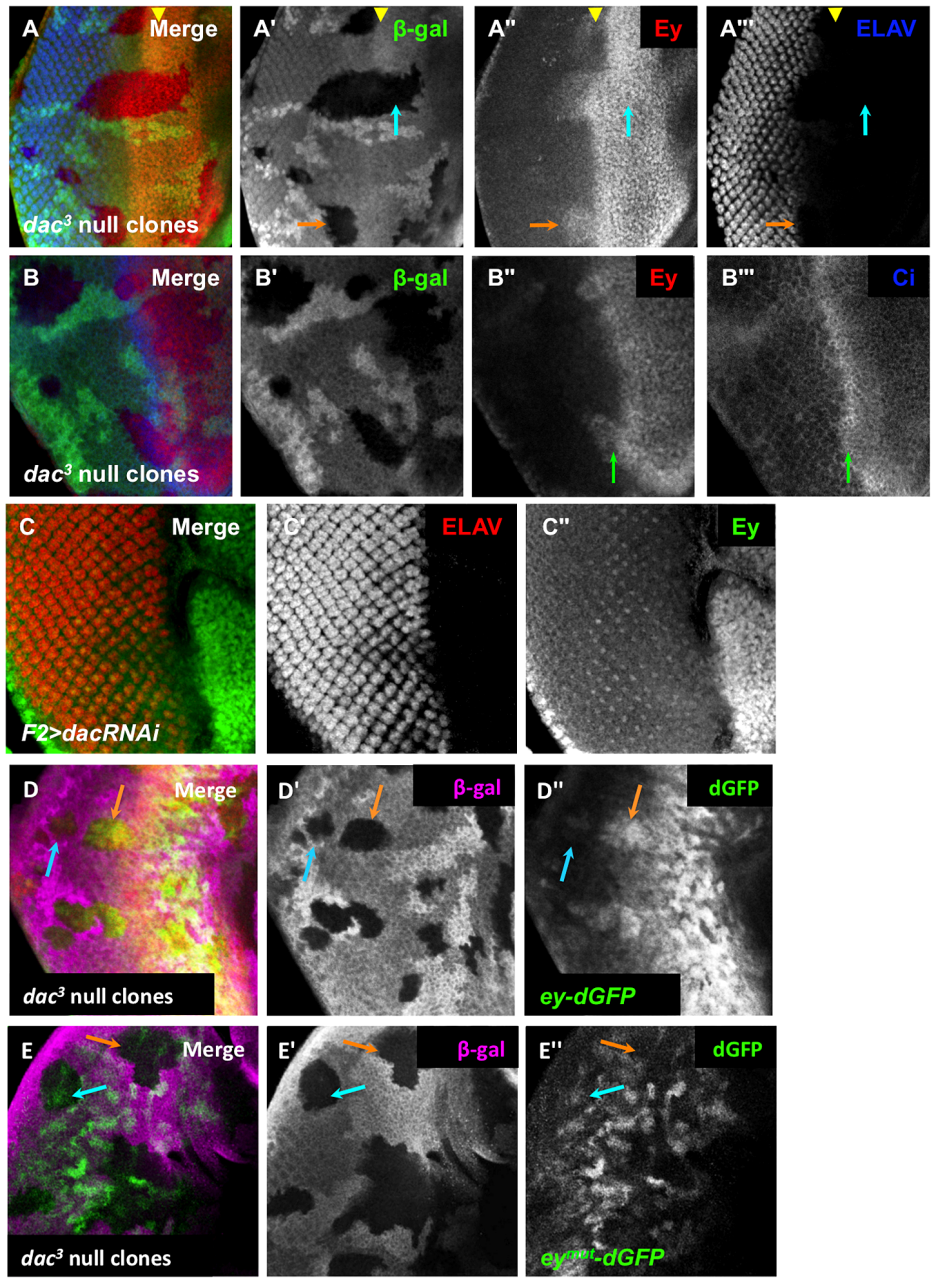
Dachshund is required for *ey* repression near the morphogenetic furrow. (A) *dac^3^* null clones, induced by *hs-flp* 48 hours after egg lay (AEL) showing β-Galactosidase, ELAV and Ey expression. The yellow arrowhead indicates the furrow. The blue arrow indicates an anterior clone. The orange arrow indicates a posterior clone. (A′) Grayscale image of β-Galactosidase expression, shown as green in A; loss of β-Galactosidase expression marks the clones. (A″) Grayscale image of Ey channel alone, red in A. (A′″) Grayscale image of ELAV channel alone showing photoreceptor differentiation, blue in A. (B) *dac^3^* null clones, induced by *hs-flp* 48 hours after egg lay (AEL) showing β-Galactosidase, Ci and Ey expression. The green arrow indicates the boundary between high and low levels of Ci. (B′) Grayscale image of β-Galactosidase expression, shown as green in B; loss of β-Galactosidase expression marks the clones. (B″) Grayscale image of Ey channel alone, red in B. (B′″) Grayscale image of Ci channel alone, blue in B. (C) *F2-Gal4* drives expression of *dacRNAi* (TRiP collection transformant ID HMS01435). Merge of ELAV and Ey expression shown. (C′) Grayscale image of ELAV expression, shown as red in C, marks differentiating photoreceptors. (C″) Grayscale image of Ey expression, shown as green in C. (D) *ey-dGFP* (green) expression in *dac^3^* null clone posterior to the morphogenetic furrow marked by loss of β-Galactosidase expression (magenta). The blue arrow indicates a clone far posterior to the furrow. The orange arrow indicates a clone posterior to but near the furrow. (D′) Grayscale image of β-Galactosidase expression in D. (D″) Grayscale image of *ey-dGFP* expression in D as revealed by anti-GFP. (E) *ey^mut^-dGFP* (green) expression in *dac^3^* null clone posterior to the morphogenetic furrow marked by loss of β-Galactosidase expression (magenta). The blue arrow indicates a clone far posterior to the furrow. The orange arrow indicates a clone near the furrow. (E′) Grayscale image of β-Galactosidase expression in E. (E″) Grayscale image of *ey^mut^-dGFP* expression in E as revealed by anti-GFP.

Within the morphogenetic furrow, we observed that Eya and So levels are not increased until after the initial decrease of Ey expression, indicating that there must be an additional mechanism that contributes to Ey negative regulation in this domain. The Ski/Sno family member Dachshund (Dac) physically interacts with Eya [20], [55], and may cooperate to regulate targets of So and Eya [28]. In mammals, the ortholog Dach interacts with the Eya and So orthologs to repress targets [56], though this interaction has not been confirmed in *Drosophila*. To test if Dac is involved in Ey repression, we generated *dac* null clones. Anterior to the furrow, Ey expression was not affected in *dac* clones, suggesting that *dac* is not required for Ey expression anterior to the morphogenetic furrow (Figure 7A). As previously reported for clones posterior to the morphogenetic furrow, we observed increased Ey expression in *dac* clones near the furrow, but not clones distant from it (Figure 7A,B) [26]. This overlaps the highest levels of Dac expression posterior to the furrow (Figure S7A,B). This shows that *dac* is required for negative regulation of Ey specifically in the domain near the morphogenetic furrow.

It is known that large *dac* clones can have delayed morphogenetic furrow progression, making it possible that Ey expression within these clones could be a secondary consequence of a delayed furrow [13]. To address this, we assayed furrow progression through small *dac* clones and compared this to the Ey expression boundary. Cubitus interruptus (Ci), the effector of Hedgehog signaling, normally accumulates to high levels in a tight band within the morphogenetic furrow, just posterior to the onset of Ey negative regulation (Figure 1A, Figure S7C). In *dac* clones spanning the furrow, Ci accumulation was not delayed, but Ey overlapped high levels of Ci, which was not observed in wild-type cells (Figure 7B, compare to Figure 1A). This result suggests that the leading edge of the morphogenetic furrow, normally correlating with Ey suppression, moves into and through these *dac* clones. As Ey suppression is delayed in these clones, it indicates that Dac is required for suppression of Ey near the furrow independent of its role in furrow progression. To further test if *dac* represses Ey posterior to the furrow, we used *F2-Gal4* to drive multiple independent *dac* RNAi transgenes, and observed that Ey expression is detected in knockdown cells posterior to the furrow (Figure 7C and data not shown). This result shows that Dac is necessary to suppress Ey expression posterior to the furrow.

We used the reporter *ey-dGFP* to determine if Dac suppresses *ey* at the transcriptional level. Like Ey, *ey-dGFP* is expressed in *dac* clones near the furrow (Figure 7D, orange arrow), but not clones far posterior to the furrow (Figure 7D, blue arrow). This indicates that Dac is required to suppress *ey* transcription near the morphogenetic furrow, consistent with the expression pattern of Dac. We also examined *ey^mut^-dGFP* in *dac* clones. First, near the morphogenetic furrow, we did not observe expression of *ey^mut^-dGFP* in *dac* clones as we had observed with *ey-dGFP* (Figure 7E, orange arrow). This result indicates that the elevated levels of wild-type reporter expression observed in *dac* clones require the So binding site. By extrapolation, this result suggests that So still activates *ey* expression in *dac* clones near the MF; this places repression by Dac earlier than suppression by So during development. In clones far posterior to the morphogenetic furrow we observed that *ey^mut^-dGFP* is expressed in *dac* clones (Figure 7E, blue arrow), suggesting the repression of the wild-type reporter observed in *dac* clones requires the So binding site. We conclude that the phenotypes of *ey* reporter expression in *dac* clones reflect regulation by So in these domains. Furthermore, we conclude that Dac suppression of *ey* expression is an earlier developmental event than repression by So.

**Figure 8:**
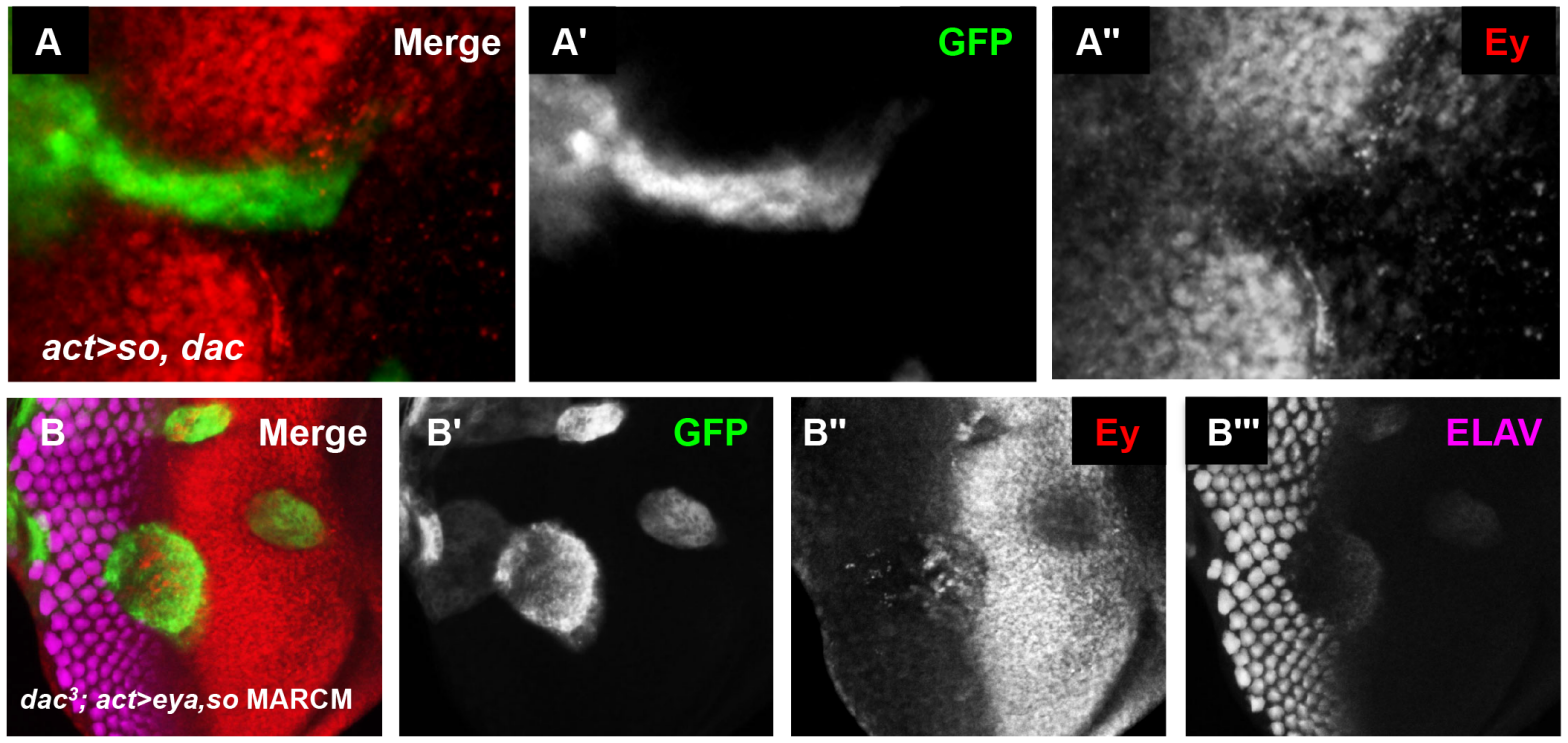
So and Eya cooperate with Dac in vivo to complete Ey repression. (A) *UAS-so* and *UAS-dac^7c4^* were co-overexpressed anterior to the furrow. (A′) Grayscale image of GFP expression in A; GFP marks the clone. (A″) Grayscale image of Ey expression in A. (B) MARCM clones that are null for *dac* while overexpressing *so* and *eya*. (B′) Grayscale image of GFP expression in B; GFP marks the clone. (B″) Grayscale image of Ey expression in B. (B″) Grayscale image of ELAV expression in B shows differentiating photoreceptors.

We next overexpressed Dac with Eya or So to see if they were sufficient to suppress Ey expression anterior to the furrow. Overexpression of *dac* or *eya* alone did not alter Ey expression (data not shown). Co-overexpression of *eya* and *dac* also had no effect on Ey expression (data not shown). However, co-overexpression of *so* with *dac* was sufficient to repress Ey expression to modest levels (Figure 8A). We conclude that Dac and So can cooperate to reduce Ey expression in vivo.

*dac* is a downstream target of the So/Eya complex in the eye [19], [25], [49]. Therefore, we wanted to determine if Ey repression anterior to the furrow by co-overexpression of Eya and So (Eya+So) requires the activation of *dac* by these genes. To test this, we generated Eya+So overexpression clones that were also null for *dac* using the MARCM technique [57]. So and Eya reduced Ey expression anterior to the furrow, though less effectively than in cells that can still express Dac (Figure 8B vs. Figure 5C). This suggests that So and Eya can repress Ey expression without Dac, but that full repression anterior to the furrow requires Dac. In MARCM clones spanning the furrow, the phenotype resembles *dac* null clones and Ey is not repressed, suggesting that Dac is specifically required in this domain (Figure 8B). Finally, in posterior clones distant from the furrow, Ey is not expressed (Figure 8B). This indicates that Eya and So are sufficient to completely suppress Ey in this domain. Together, these results indicate that Dac is required near the morphogenetic furrow to negatively regulate Ey expression, but that So and Eya can cooperate to repress Ey independent of Dac further posteriorly.

## Discussion

In this work, we have found that a switch from high to low levels of Ey expression is required for normal differentiation during retinal development. We also present a mechanism of Ey regulation by the RD gene network members Eya, So, and Dac. Specifically, we report that So switches from being an activator to a suppressor of *ey* expression, both depending on a So binding site within an *ey* eye-specific enhancer. We additionally report that the So cofactors Eya and Dac are required for *ey* repression posterior to the furrow but not for its maintenance ahead of the furrow, and are sufficient to cooperate with So to mediate Ey repression within the normal Ey expression domain.

Our results support a Gro-independent mechanism for the suppression of target gene expression by the transcription factor Sine oculis (So). An independent study has also shown that So can repress the selector gene *cut* in the antenna in a Gro-independent process though the mechanism was not determined [46]. We observe that Ey is expressed at low levels posterior to the morphogenetic furrow. However, when *so* expression is lost in clones posterior to the furrow, Ey expression and *ey-dGFP* expression are strongly activated. We show that this is not simply a default response of *ey* to So loss, as removing So from developmentally earlier anterior cells results in reduced *ey* expression. We also observe that knockdown of So specifically in differentiating cells using RNAi causes a similar phenotype, suggesting that an activator of Ey expression is expressed in differentiating photoreceptors. Mutation of a known So binding site in *ey-dGFP* results in activation of the reporter posterior to the furrow, supporting a model that binding of So to the enhancer prevents inappropriate activation of *ey* expression posterior to the furrow. Finally, in vitro we observe that an excess of So is sufficient to prevent activation of the enhancer and observe that in vivo overexpression of So can suppress normal Ey expression. Our observations are consistent with what in vitro studies have indicated about So function: when So binds DNA without Eya, it can only weakly activate transcription [45, and this work]. However, our work introduces a novel mechanism of regulation for So targets, in which So occupancy of an enhancer prevents other transcription factors from inducing high levels of target gene expression. Our results also indicate that suppression of robust *ey* expression is an important developmental event. It is not yet clear if maintaining basal expression of *ey*, rather than completely repressing it, is developmentally important; however, it is possible that the ultimate outcome of a basal level of *ey* transcription may be necessary for the completion of retinal development [58].

Our results also show that *eya* is required for Ey suppression in vivo. However, consistent with its characterization as a transcriptional coactivator, our in vitro analysis does not indicate a direct role for Eya in repression. Previous studies, and our observations, indicate that Eya is required for the expression of So posterior to the furrow in the third instar [18], [24], [25], [36], and Figure S5. Additionally, our reporter analysis shows that Eya regulation of *ey* requires the So binding site. We propose that the simplest model for Eya function in the suppression of *ey* is through its established function as a positive regulator *of* So expression, as we observe that overexpression of So alone is sufficient to weakly repress Ey expression and to block reporter activation in vitro. This model could also account for the results reported by us and others regarding the inability of this *UAS-so* construct to induce ectopic eye formation [16], [36], [46], [59]. Briefly, the primary function of So in ectopic eye formation is to repress the non-eye program [46]. Overexpressing the So construct used in this study alone is not sufficient to induce this program, possibly because the transgene expression level is not sufficient; however, co-expression of the *so* positive regulator Eya is sufficient to induce robust ectopic eye formation [16], [36]. In light of our findings, we propose that Eya co-expression is necessary to induce So expression to sufficient levels to block transcriptional activation of non-eye targets to permit the induction of the ectopic eye program; however we cannot rule out that other functions of Eya may play a role.

We further demonstrate that *dac* expression is required specifically near the furrow for Ey repression. In addition, we show that the So binding site is required for strong *ey* expression in *dac* clones near the furrow, suggesting that So activates *ey* in these clones. This suggests that repression by Dac occurs before the transition to repression by So, making Dac the first repressor of *ey* expression at the furrow, and identifying how the initiation of repression occurs before So levels increase. We further show that Eya and So are sufficient to repress *ey* expression in *dac* mutant clones anterior to the furrow, though not as completely as in cells that express Dac. This result indicates that Dac is not an obligate partner with Eya and So in *ey* repression, but is required for the full suppression of *ey*. One model would be that Dac and So can cooperate in a complex to modestly repress *eyeless* directly. This would be consistent with our loss-of-function and reporter data as well as the observation that Dac and So misexpression can weakly cooperate to repress Ey anterior to the furrow. However, while a similar complex has been described in mammalian systems, previous studies have been unable to detect this physical interaction in *Drosophila* [44], [45], [55], [60]. An alternative model is that Dac suppresses *ey* expression indirectly and in parallel to Eya and So. A previous study has shown that *dac* expression is necessary and sufficient near the furrow to inhibit the expression of the zinc finger transcription factor Teashirt (Tsh) [26]. Tsh overlaps Ey expression anterior to the furrow, and can induce Ey expression when misexpressed [42], [43]. Furthermore, *tsh* repression is required for morphogenetic furrow progression and differentiation [42], [43]. In light of these previously published findings, we propose that a simpler model based on current knowledge is that Dac repression of *tsh* at the morphogenetic furrow reduces Ey expression indirectly. Future studies may distinguish between these mechanisms.

In addition to the role of the RD gene network in *ey* modulation, we identify that signaling events within the morphogenetic furrow indirectly regulate the switch to low levels of *ey* expression. It has been shown that signaling pathways activated in the morphogenetic furrow increase levels of Eya, So and Dac; furthermore, it is proposed that this upregulation alters their targets, creating an embedded loop within the circuitry governing retinal development and allowing signaling events to indirectly regulate targets through the RD network [26], [28], [61]. The identification of *ey* regulation by So posterior to the morphogenetic furrow represents a direct target consistent with this model.

**Figure 9:**
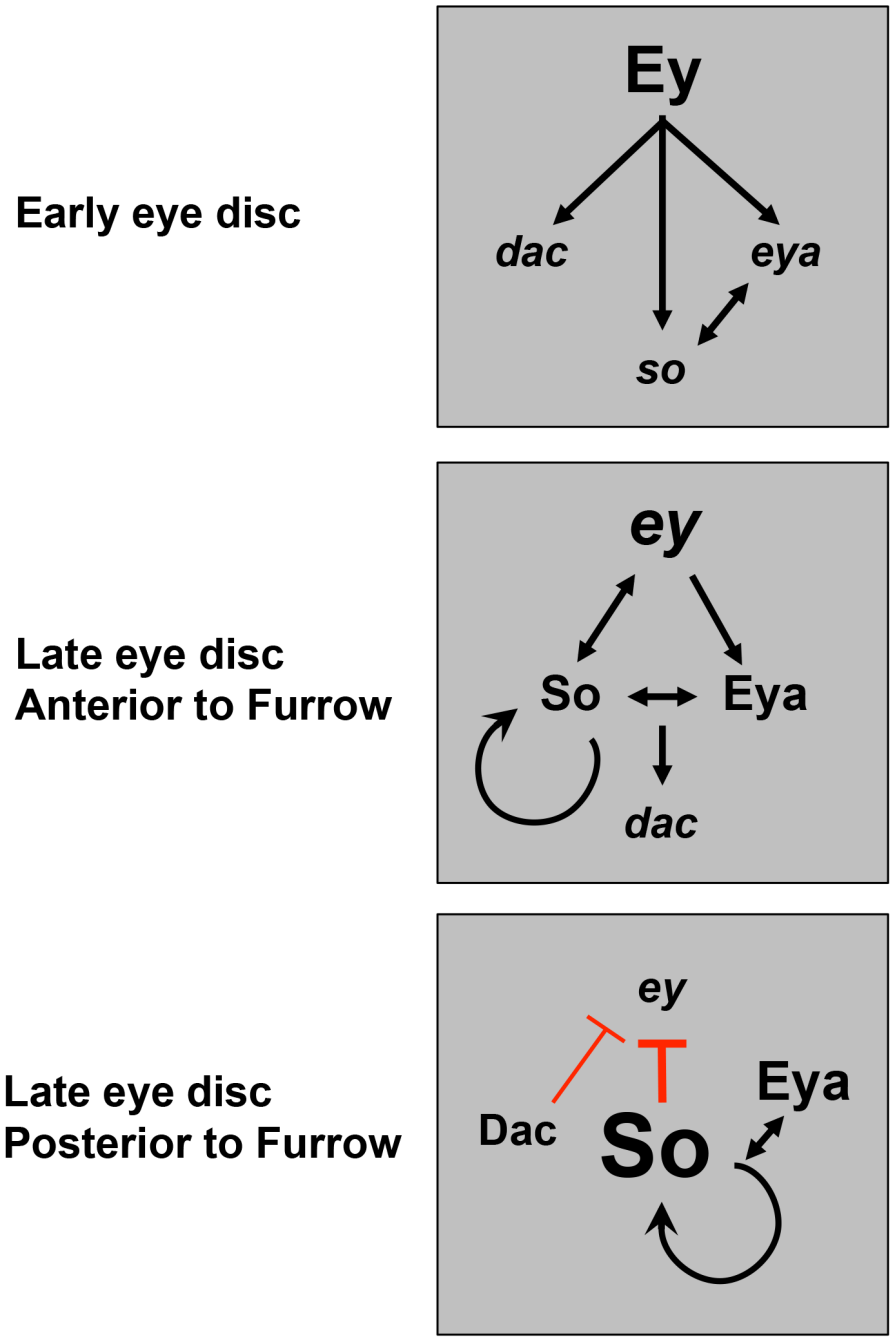
A model for dynamic RD gene network interactions during the third instar. Previous studies have shown that prior to the third instar, Ey activates expression of downstream RD genes. This work shows that anterior to the furrow during third instar, positive feedback loops are maintained among the RD network members, with So feeding back to help promote and maintain *ey* expression. Just posterior to the morphogenetic furrow, Dac represses *ey* transcription. Finally, posterior to the furrow, high levels of So, induced by Eya, are sufficient to prevent activation of high levels of *ey* transcription.

In conclusion, we present a model that rewiring of the RD network activates different dominant sub-circuits to drive key transitions in development (Figure 9). To the interactions previously identified by others, we add that strong upregulation of So, dependent on Eya, results in minimal levels of *ey* transcription [18], [25]. We propose that the identification of this novel sub-circuit of the RD network provides a mechanism for terminating the self-perpetuating loop of determination associated with high levels of Ey, permitting the onset of differentiation and the completion of development. Together, these results give us a new view into how temporal rewiring within the RD network directs distinct developmental events.

## Materials and Methods

### Generation of destabilized GFP (dGFP) constructs for in vivo experiments

#### pH-dGFP-attB

A 285 bp *φC31 attB* fragment was PCR-amplified from *p[ACMAN]* [39], cut with AatII, and cloned into *pH-Stinger* [41], resulting in the construct *pH-Stinger-attB*. *dGFP* encodes a destabilized variant of enhanced green fluorescent protein, amplified from *10XSTAT92E-GFP* [62] with 5′ AgeI and 3′ NotI tails and cloned into *pH-Stinger-attB*, generating *pH-dGFP-attB*.

#### *ey-dGFP* and *ey^mut^-dGFP*

*ey-dGFP* was generated by PCR on genomic DNA of wild-type flies by using the following primer sets: 5′-CGGAATTCCAAGTACAAACTGACTTCTTG-3′; 5′-CGCGGATCCGAATTCGAGAAATATCACATGGCC-3′. 5′ EcoRI and 3′ BamHI sites were added and used for subcloning into *pH-dGFP-attB*. The So site was mutated by changing GAG to CCC and introduced by two-step PCR to generate *ey^mut^-dGFP* [22].

#### UAS-dGFP

To generate the *UAS-dGFP* construct, *dGFP* was first amplified from the *10XSTAT92E-GFP* construct with XbaI and XhoI tails. PCR product was then digested and ligated into *pUAST-attB* vector (a gift from Konrad Basler). Positive clones were sequenced to confirm sequence integrity and orientation.

For transgenic fly generation, each construct was injected into a docking site at 68A (P2). Correct integration events were identified by genomic PCR by standard methods [22], [39].

### Generation of *ey-Luc*, *ey^short^-Luc*, and *ey^mut^-Luc*

The enhancer sequences were amplified from *ey-dGFP* or *ey^mut^-dGFP* with XhoI and NheI tails. PCR fragments were digested and ligated per the manufacturer's instructions (NEB, Takara) directionally into pGL3-Basic (Promega). Correct ligation events were identified by sequencing to generate *ey-Luc* and *ey^mut^-Luc*, respectively. *ey^short^-Luc* was amplified from *ey-Luc* and generates a truncated enhancer that ends 8 bp upstream of the So binding site.

### S2 cell culture, transfection and luciferase assays

*Drosophila* S2 cells were cultured in Schneider's medium containing 10% fetal bovine serum and antibiotics. Cells were transiently transfected in 48-well plates using Cellfectin (Invitrogen) according to the manufacturer's protocol. Cells were transfected with *ey*-*Luc*, *ey^short^-Luc*, or *ey^mut^-Luc*, in the presence or absence of Eya and So in pMT vector (Invitrogen, a gift from Ilaria Rebay), along with tub-Renilla luciferase in pRL vector (a gift from K Basler). 24 hrs after transfection, cells were induced with CuSO_4_ at a final concentration of 500 µM. Luciferase activity was assayed 2 days after induction using the Dual-Glo kit (Promega) according to the manufacturer's protocol. Data were graphed in GraphPad Prism and labeled using Adobe Illustrator.

### Crosses and fly husbandry

For a list of the genotypes used, please reference Table S1. All crosses were performed on standard cornmeal agar at 25°C unless otherwise noted. Heat shocks were performed at 37°C. Flipout-Gal4 [63] crosses were heat shocked for 8 min, 48 hrs after egg laying (AEL). For loss-of-function clones or MARCM clones [57], heat shocks were performed for 1 hr at 48 hrs AEL, or, for *so^3^* and *eya^cliIID^* clones, 72 hrs AEL. Wandering third instar larvae were collected and dissected using standard methods as previously described [37].

### Immunohistochemistry

Staining was performed as previously described [64]. For antibodies used, please reference Table S2.

### Microscopy and Image processing

Imaginal disc images were captured using a Zeiss LSM confocal microscope. LSMs were stacked using ImageJ software and stacks were merged in ImageJ and prepared for figures using Adobe Photoshop. Staining quantification for Eya, Ey and So: orthogonal sections were generated using ImageJ and represent approximately 10 micron wide slices through the full depth of the disc (n = 5); sections were resliced in ImageJ to generate XZ stacks which were summed. The apical ROI was measured based on the width of the Eya signal in photoreceptors. The basal ROI was the same ROI, shifted basally to exclude the apical Eya signal. Pixel intensity was calculated using the plot profile function, and values were normalized. Pixel intensity plots and bar graph of average fold change were generated in GraphPad Prism. For adult images, adults were frozen at −80°C for 30 minutes. Light microscopy images of adult heads were captured on a Zeiss Axioplan microscope, and were processed with Adobe Photoshop software.

## Supporting Information

Figure S1 Consequence of Ey overexpression posterior to the morphogenetic furrow. (A) *w^1118^* eye disc showing expression of Sens (alone in A′), Ey (alone in A″), and ELAV (alone in A′″). (B) *GMR-Gal4* driving expression of ectopic Ey expression from the *UE10* transgene (*GMR>ey*) showing expression of Sens (alone in B′), Ey (alone in B″), and ELAV (alone in B′″). (C) *Lz-Gal4* driving expression of ectopic Ey expression (*Lz>ey*) from the *UE10* transgene showing expression of Sens (alone in C′), Ey (alone in C″), and ELAV (alone in C′″) (D) *CantonS* (CS) adult eye. (E) Adult eye of *Lz>ey* animal. (F) Resin section through adult *CS* eye (G) Resin section through adult *Lz>ey* eye.
(TIF)

Figure S2 Loss of *so* expression leads to Ey reactivation posterior to the furrow. (A) *so^3^* null clones, induced by hs-flp 72 hrs AEL. (B) Orthogonal section through the largest clone near the furrow (B′) Grayscale image of Ey expression, red in A,B. (B″) Grayscale image of So, green in A,B; loss of So expression marks the clones. (B′″) Grayscale image of ELAV expression, blue in A,B, marks differentiating photoreceptors. (C) *so^3^* null clones, induced by hs-flp 72 hrs AEL, full stack showing Lamin and Ey expression. (C′) single optical section of C. (C″) Nuclear lamin expression, red in C,C′. (C′″) Ey expression, green in C, C′. (D) *F2-Gal4* drives expression of *UAS-dGFP*. (D′) Grayscale image of GFP expression, green in D. (D″) Grayscale image of Sens expression, red in D; Sens marks R8 photoreceptors. (D′″) Grayscale image of Eya expression, magenta in D. (E) *F2-Gal4* drives expression of *soRNAi* (VDRC transformant KK108128). (E′) Grayscale image of Eya expression, green in E. (E″) Grayscale image of Ey expression, red in C. (E′″) Grayscale image of So, magenta in E. (F) Driving *UAS-dGFP* with *F2-Gal4* does not disrupt normal eye development, resulting in a normal size eye with regular ommatidial facets. (G) *F2>soRNAi* has a slightly smaller, mild rough eye phenotype.
(TIF)

Figure S3 Expression of *ey-dGFP* and *ey^mut^-dGFP* is dynamic. For clarity, individual channels for each panel of Figure 1A–F are shown. For all panels, Senseless expression initiates at the furrow and is shown in red as a reference. Reporter expression (*ey-dGFP* or *ey^mut^-dGFP* as indicated), revealed by anti-GFP staining is shown in green. The terms early, mid and late refer to MF progression during the third instar. Representative discs shown that were age matched as close as possible based on columns of Sens positive cells. (A–A″) Merge and individual channels for the disc shown in Figure 3A. (B–B″) Merge and individual channels for the disc shown in Figure 3B. (C–C″) Merge and individual channels for the disc shown in Figure 3C. (D–D″) Merge and individual channels for the disc shown in Figure 3D. (E–E″) Merge and individual channels for the disc shown in Figure 3E. (F–F″) Merge and individual channels for the disc shown in Figure 3F.
(TIF)

Figure S4 *gro* is not required for Ey repression. Null loss-of-function clones were generated for *gro*; Ey expression was not affected in either anterior or posterior clones (A–A″).
(TIF)

Figure S5 *eya* knockdown using *F2-Gal4*. (A) *F2-Gal4* drives expression of *eyaRNAi* (VDRC transformant KK108071). (A′) Grayscale image of Eya expression, green in A. (A″) Grayscale image of Ey expression, red in A. (A′″) Grayscale image of So, magenta in A. (B). RNAi knockdown of *eya* driven by *F2-Gal4* results in a rough eye. (Control in Figure S1F).
(TIF)

Figure S6 Flipout-Gal4 driving *eya* and *so* expression at 18°C. (A) *Flipout-Gal4* was used to co-express *UAS-so*, *UAS-eya*, and *UAS-GFP*. Crosses were raised at 18°C (A′) Grayscale image of GFP expression, green in A; GFP marks the clone (A″) Grayscale image of Ey expression, red in A. White arrow indicates ectopic Ey in the antennal field.
(TIF)

Figure S7 Expression of Ci and Dac relative to Ey in the furrow. (A) Ey and Dac expression in a *w^1118^* third instar eye-antennal imaginal disc; yellow line indicates site of orthogonal section shown in B (A′) Dac expression, green in A. (A″) Ey expression, red in A. (B–B″) Orthogonal sections of A–A″. (C–C′″) Orthogonal section of disc shown in figure 1A (C) Merge. (C′) Ey expression, red in C. (C″) Ci expression, green in C. (C″) Sens expression, magenta in C.
(TIF)

Table S1 Fly stocks used and/or generated in this report. Fly stocks are listed. If the genotype is ambiguous concerning the chromosomal location of a transgene or if a specific integration site is known, this is indicated in the field “Chrom.” Specific integration sites are indicated in parentheses. Stock sources or references are also provided.
(DOCX)

Table S2 Antibodies used in this study. Antigen, host, dilution and source are indicated.
(DOCX)
