# Supplementary figures and images for: Dynamic Rewiring of the Drosophila Retinal Determination Network Switches Its Function from Selector to Differentiation
Source: PLoS Genet. 2013 Aug 29;9(8):e1003731. doi: 10.1371/journal.pgen.1003731 (PMC3757064; doi:10.1371/journal.pgen.1003731)

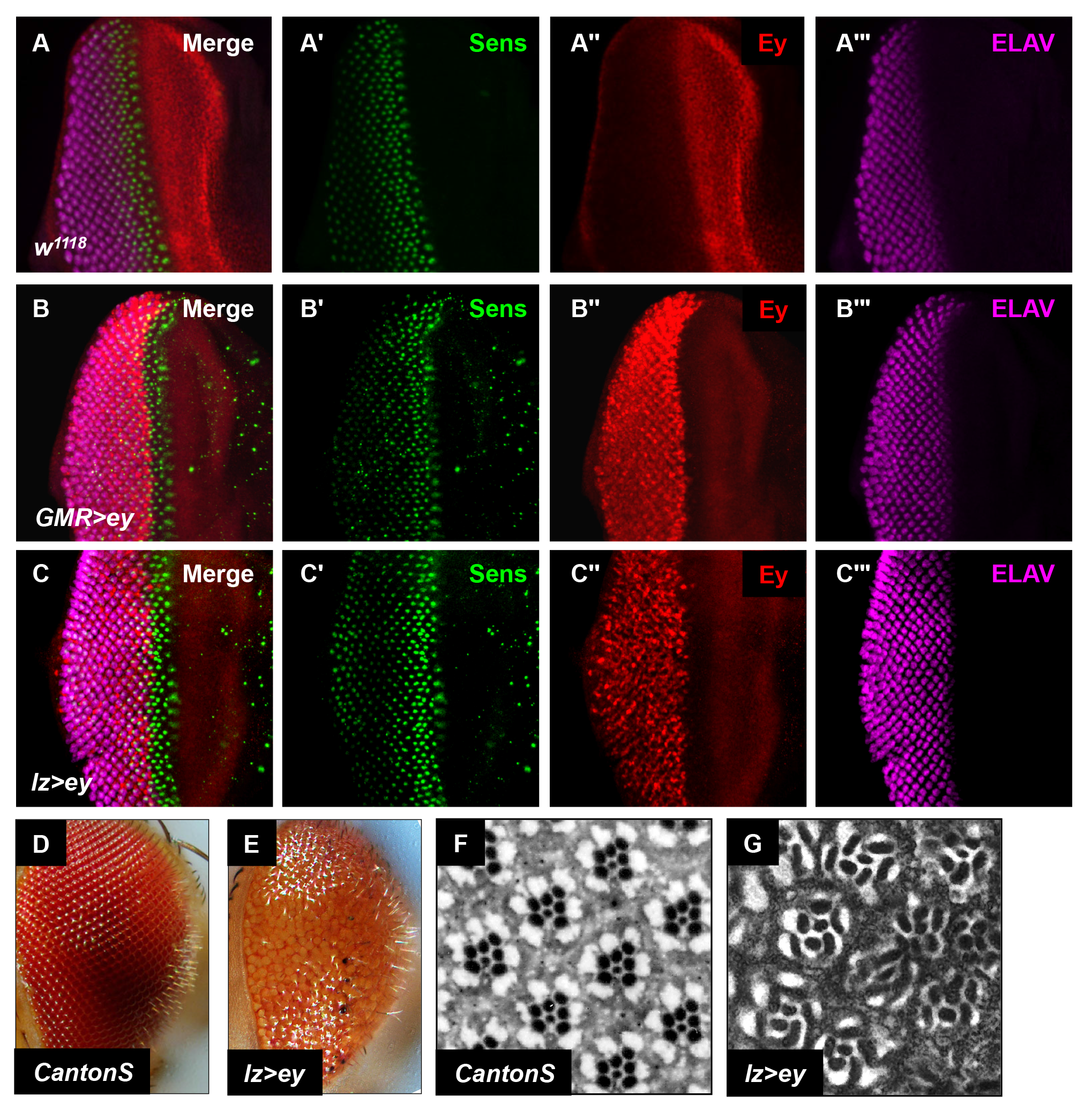

Supplement: Figure S1 — Consequence of Ey overexpression posterior to the morphogenetic furrow. (A) w1118 eye disc showing expression of Sens (alone in A′), Ey (alone in A″), and ELAV (alone in A′″). (B) GMR-Gal4 driving expression of ectopic Ey expression from the UE10 transgene (GMR>ey) showing expression of Sens (alone in B′), Ey (alone in B″), and ELAV (alone in B′″). (C) Lz-Gal4 driving expression of ectopic Ey expression (Lz>ey) from the UE10 transgene showing expression of Sens (alone in C′), Ey (alone in C″), and ELAV (alone in C′″) (D) CantonS (CS) adult eye. (E) Adult eye of Lz>ey animal. (F) Resin section through adult CS eye (G) Resin section through adult Lz>ey eye. (TIF) [file pgen.1003731.s001.tif]

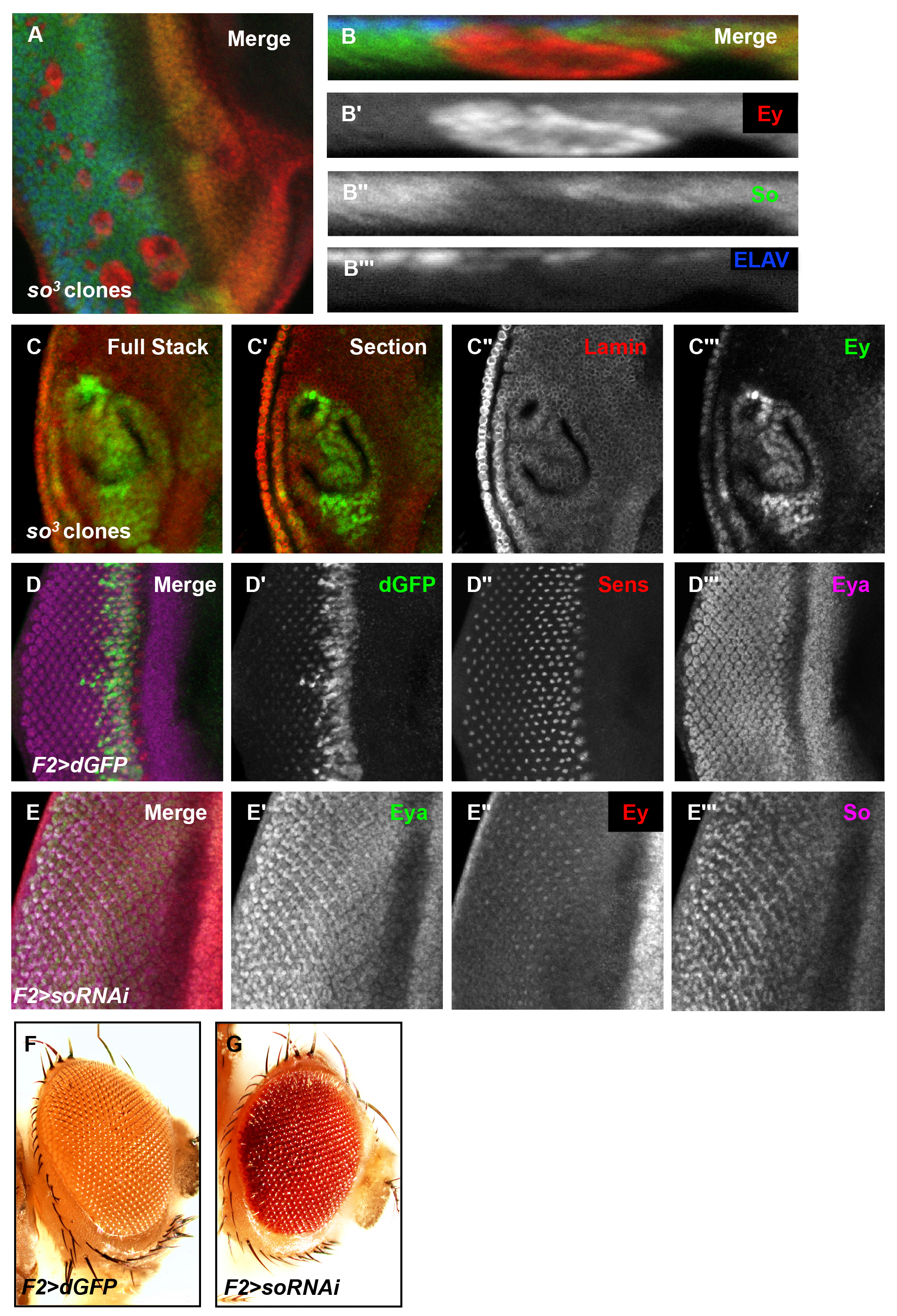

Supplement: Figure S2 — Loss of so expression leads to Ey reactivation posterior to the furrow. (A) so3 null clones, induced by hs-flp 72 hrs AEL. (B) Orthogonal section through the largest clone near the furrow (B′) Grayscale image of Ey expression, red in A,B. (B″) Grayscale image of So, green in A,B; loss of So expression marks the clones. (B′″) Grayscale image of ELAV expression, blue in A,B, marks differentiating photoreceptors. (C) so3 null clones, induced by hs-flp 72 hrs AEL, full stack showing Lamin and Ey expression. (C′) single optical section of C. (C″) Nuclear lamin expression, red in C,C′. (C′″) Ey expression, green in C, C′. (D) F2-Gal4 drives expression of UAS-dGFP. (D′) Grayscale image of GFP expression, green in D. (D″) Grayscale image of Sens expression, red in D; Sens marks R8 photoreceptors. (D′″) Grayscale image of Eya expression, magenta in D. (E) F2-Gal4 drives expression of soRNAi (VDRC transformant KK108128). (E′) Grayscale image of Eya expression, green in E. (E″) Grayscale image of Ey expression, red in C. (E′″) Grayscale image of So, magenta in E. (F) Driving UAS-dGFP with F2-Gal4 does not disrupt normal eye development, resulting in a normal size eye with regular ommatidial facets. (G) F2>soRNAi has a slightly smaller, mild rough eye phenotype. (TIF) [file pgen.1003731.s002.tif]

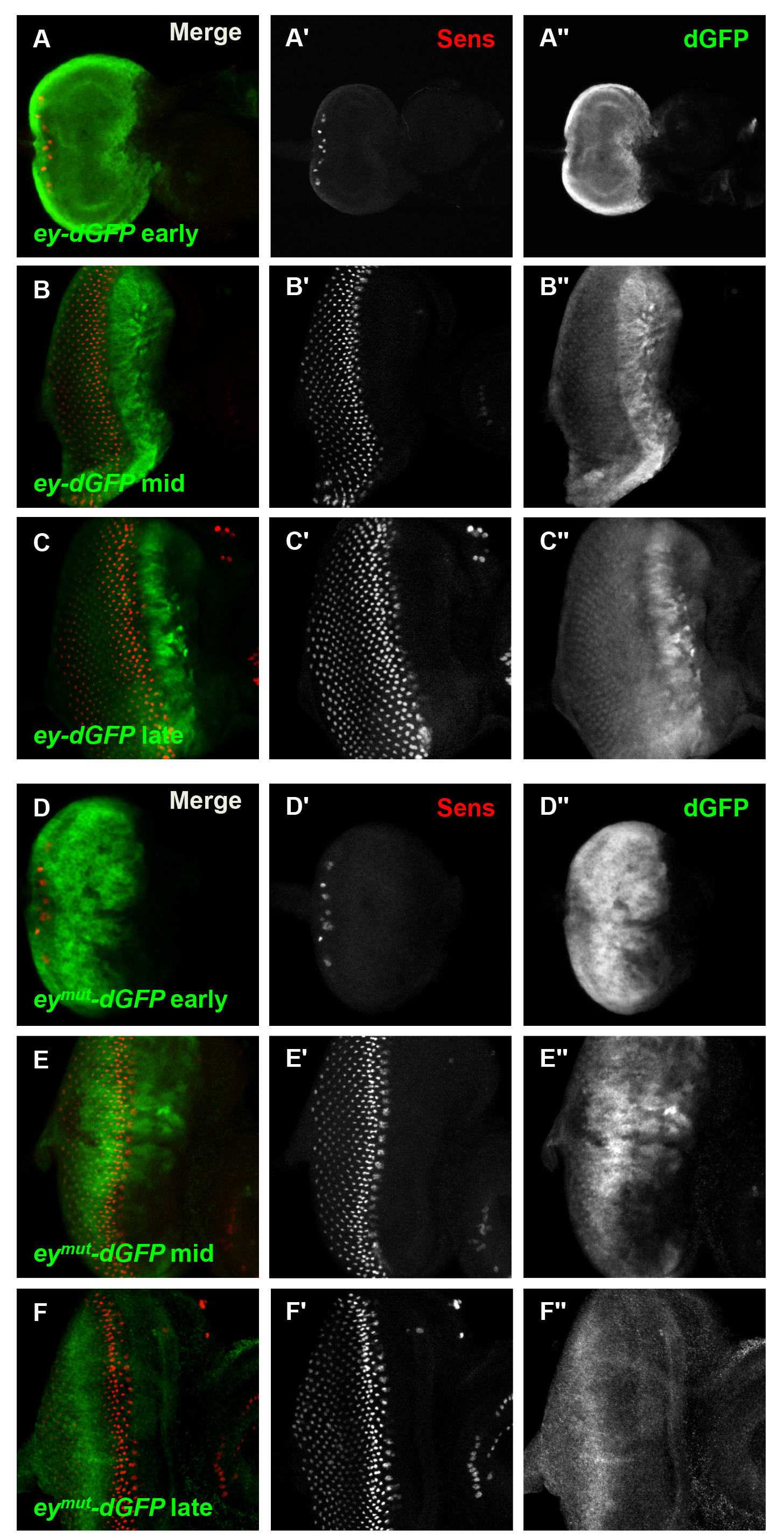

Supplement: Figure S3 — Expression of ey-dGFP and eymut-dGFP is dynamic. For clarity, individual channels for each panel of Figure 1A–F are shown. For all panels, Senseless expression initiates at the furrow and is shown in red as a reference. Reporter expression (ey-dGFP or eymut-dGFP as indicated), revealed by anti-GFP staining is shown in green. The terms early, mid and late refer to MF progression during the third instar. Representative discs shown that were age matched as close as possible based on columns of Sens positive cells. (A–A″) Merge and individual channels for the disc shown in Figure 3A. (B–B″) Merge and individual channels for the disc shown in Figure 3B. (C–C″) Merge and individual channels for the disc shown in Figure 3C. (D–D″) Merge and individual channels for the disc shown in Figure 3D. (E–E″) Merge and individual channels for the disc shown in Figure 3E. (F–F″) Merge and individual channels for the disc shown in Figure 3F. (TIF) [file pgen.1003731.s003.tif]

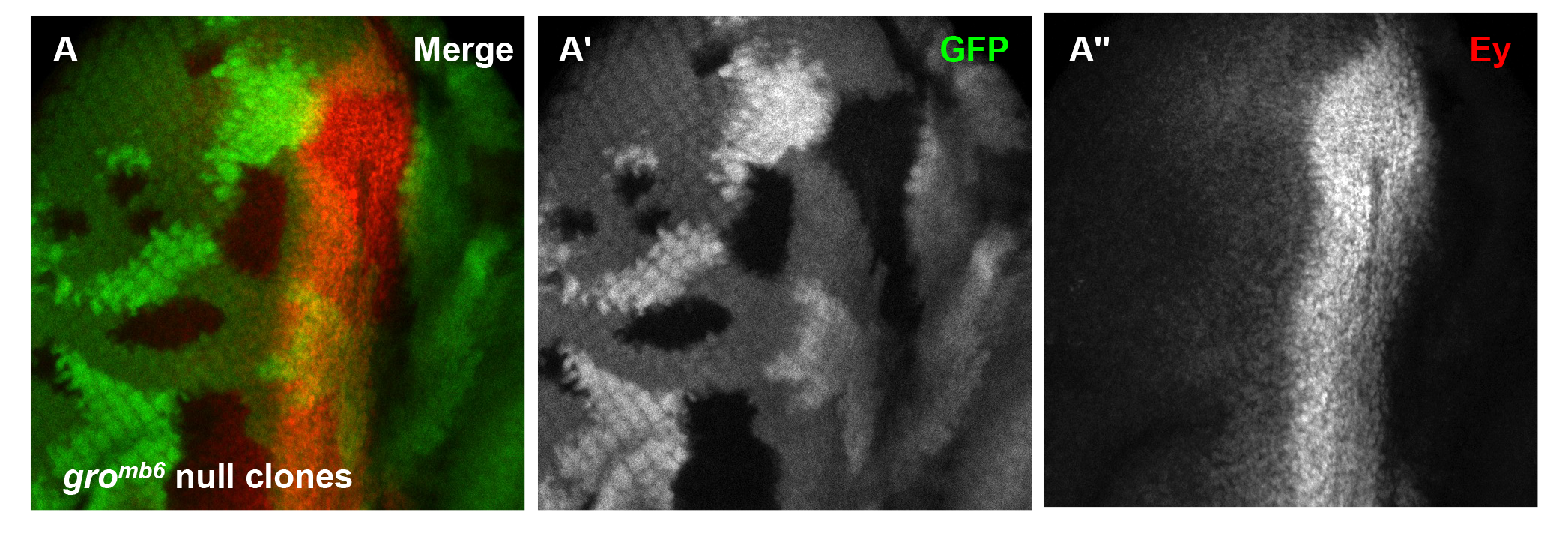

Supplement: Figure S4 — gro is not required for Ey repression. Null loss-of-function clones were generated for gro; Ey expression was not affected in either anterior or posterior clones (A–A″). (TIF) [file pgen.1003731.s004.tif]

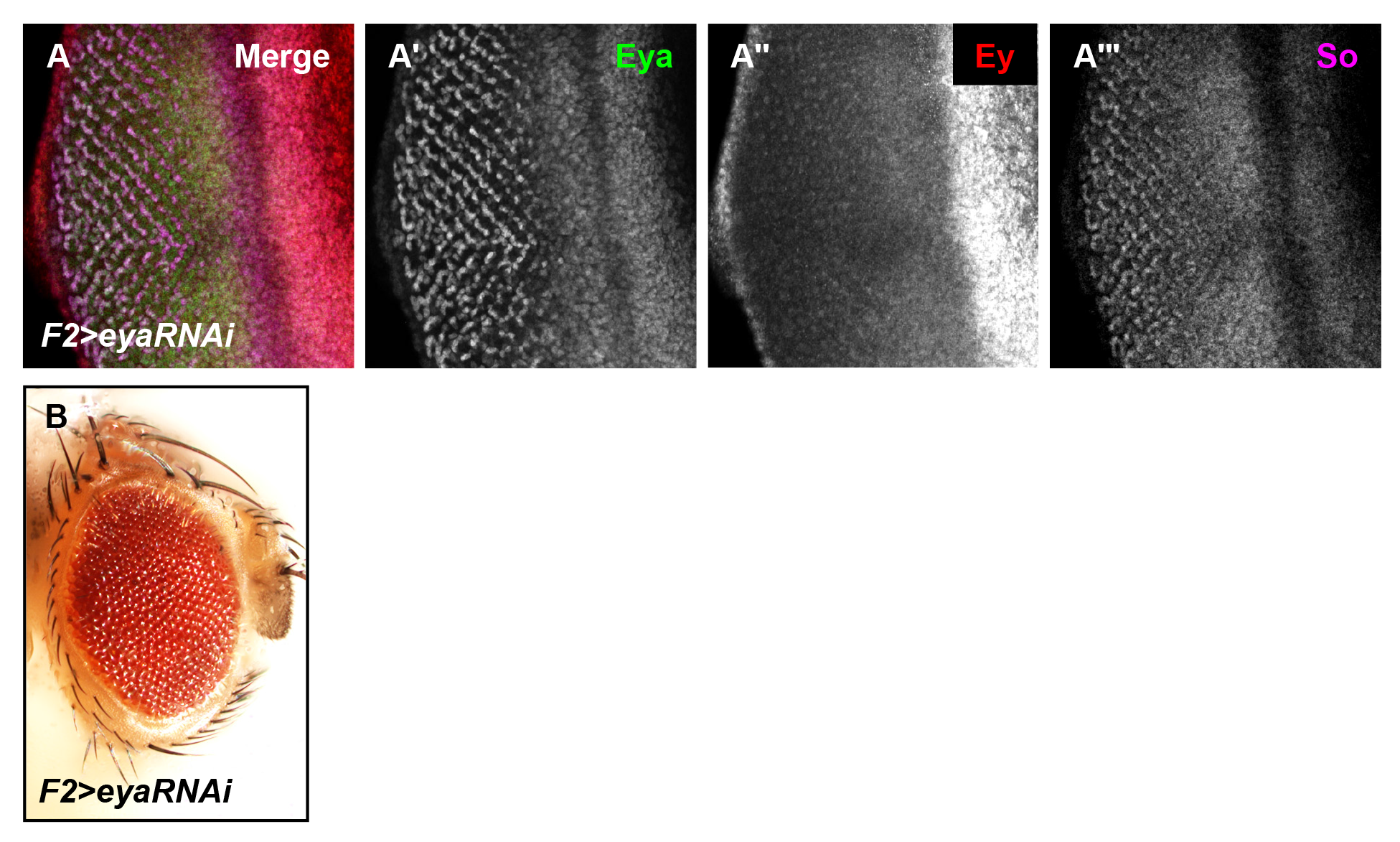

Supplement: Figure S5 — eya knockdown using F2-Gal4. (A) F2-Gal4 drives expression of eyaRNAi (VDRC transformant KK108071). (A′) Grayscale image of Eya expression, green in A. (A″) Grayscale image of Ey expression, red in A. (A′″) Grayscale image of So, magenta in A. (B). RNAi knockdown of eya driven by F2-Gal4 results in a rough eye. (Control in Figure S1F). (TIF) [file pgen.1003731.s005.tif]

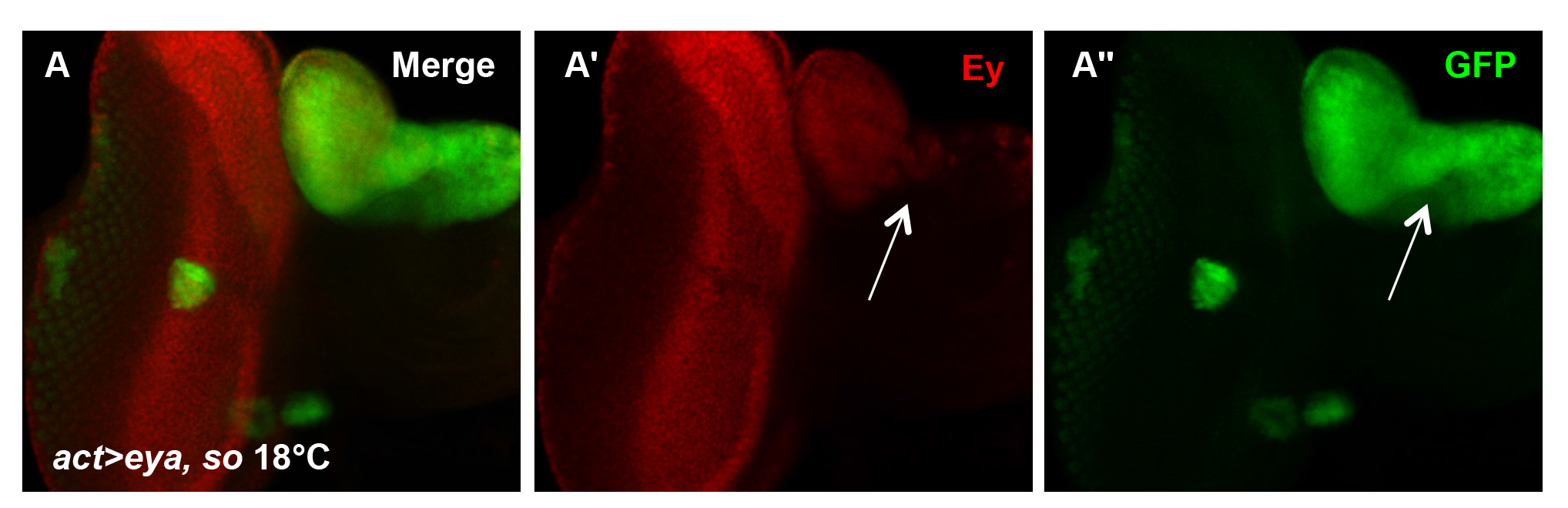

Supplement: Figure S6 — Flipout-Gal4 driving eya and so expression at 18°C. (A) Flipout-Gal4 was used to co-express UAS-so, UAS-eya, and UAS-GFP. Crosses were raised at 18°C (A′) Grayscale image of GFP expression, green in A; GFP marks the clone (A″) Grayscale image of Ey expression, red in A. White arrow indicates ectopic Ey in the antennal field. (TIF) [file pgen.1003731.s006.tif]

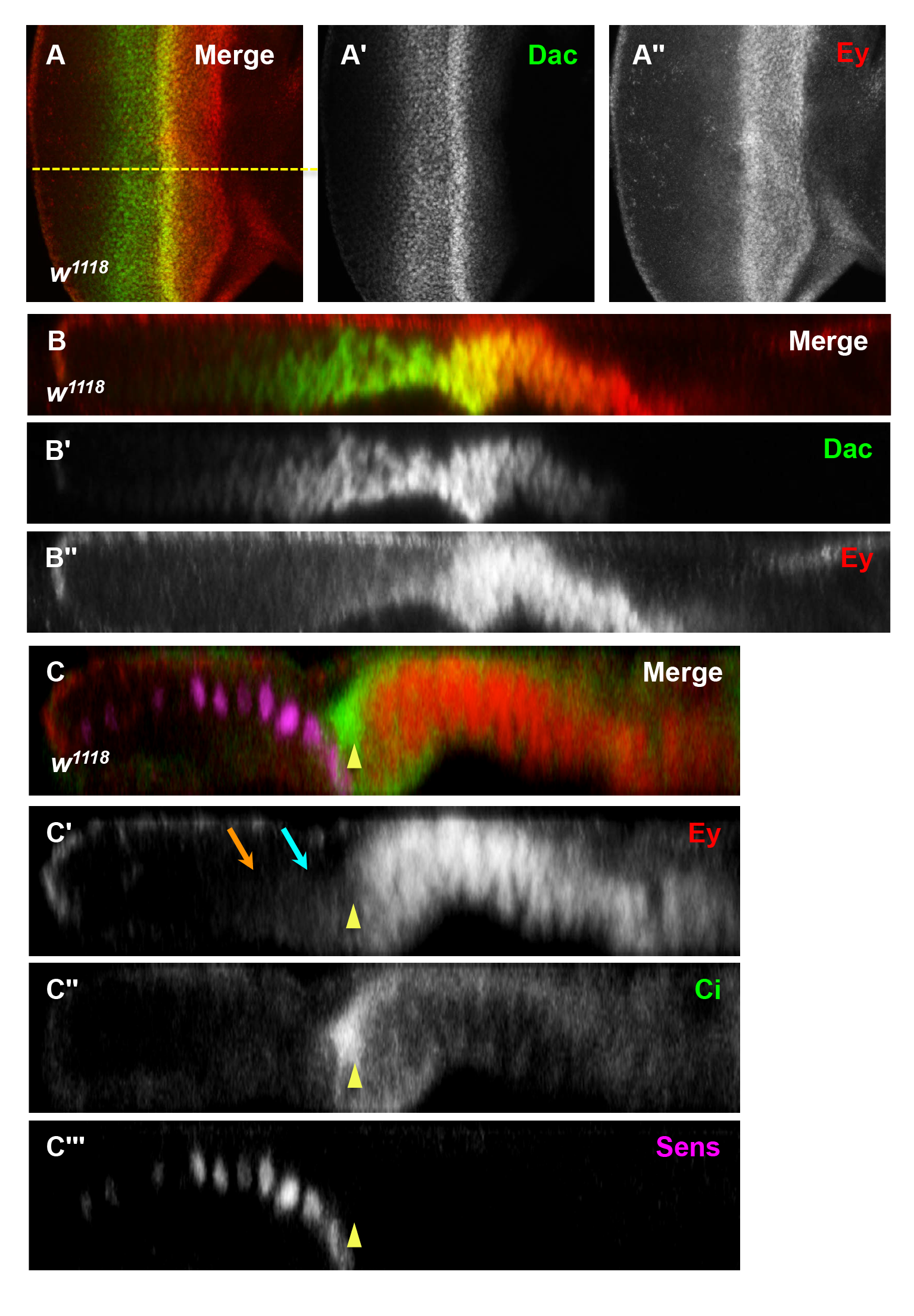

Supplement: Figure S7 — Expression of Ci and Dac relative to Ey in the furrow. (A) Ey and Dac expression in a w1118 third instar eye-antennal imaginal disc; yellow line indicates site of orthogonal section shown in B (A′) Dac expression, green in A. (A″) Ey expression, red in A. (B–B″) Orthogonal sections of A–A″. (C–C′″) Orthogonal section of disc shown in figure 1A (C) Merge. (C′) Ey expression, red in C. (C″) Ci expression, green in C. (C″) Sens expression, magenta in C. (TIF) [file pgen.1003731.s007.tif]
