# Supplementary material for: Dynamic Rewiring of the Drosophila Retinal Determination Network Switches Its Function from Selector to Differentiation
Source: PLoS Genet. 2013 Aug 29;9(8):e1003731. doi: 10.1371/journal.pgen.1003731 (PMC3757064; doi:10.1371/journal.pgen.1003731)
Supplement: Table S1 — Fly stocks used and/or generated in this report. Fly stocks are listed. If the genotype is ambiguous concerning the chromosomal location of a transgene or if a specific integration site is known, this is indicated in the field “Chrom.” Specific integration sites are indicated in parentheses. Stock sources or references are also provided. (DOCX) [file pgen.1003731.s008.docx]

**Supplemental Table 1. Fly stocks used and/or generated in this report.**

| **Genotype** | **Chrom** | **Source/Reference** |
| --- | --- | --- |
| *w^1118^* |  | Bloomington |
| *ywhsflp; act>CD2>Gal4, UAS-GFP(nls)/CyO; MKRS/TM6B* |  | Georg Halder |
| *w; F2-GAL4* | 3 | Pepple, et. al., 2008 |
| *hsflp; FRT42D arm-lacZ M* |  | Mardon |
| *hsflp; Ubi-GFP FRT40A* |  | Mardon |
| *hsflp; FRT82 Ubi-GFP* |  | Mardon |
| *tubGAL80, FRT19A; eyFLP5,act>y+>GAL4,UAS-GFP* |  | Tian Xu |
| *UAS-CD8::GFP, hsflp, w; tub-Gal80 FRT40A; tub-GAL4/TM6b* |  | Hugo Bellen |
| *w; FRT42D so^3^/ CyO* |  | Mardon |
| *w; eya^cliIId^ FRT40A/ CyO* |  | Mardon |
| *w; dac^3^ FRT40A/ CyO* |  | Mardon |
| *w; FRT42D ptc^S2^/CyO* |  | Mardon |
| *w; smo^3^, mad^1-2^ FRT40A/CyO* |  | Mlodzik |
| *w; N^54/9^ FRT19A* |  | Bellen (Baonza and Freeman, 2001) |
| *w; FRT82 gro^mb6^* |  | Shvartsman (Jennings, et.al.,2008) |
| *w; UAS-dGFP* | 3(68A) | This study |
| *w; UAS-NICD* | 3 | Rui Chen |
| *w; UAS-ci[rep]* | 2 | T. Kornberg |
| *w; UAS- Dl* | 2 | Hamed Jafar-Nejad |
| *w; UAS-hh[m4]* | 3 | Mardon |
| *w; UAS-tkv^act^* | 3 | Tetsuo Tabata |
| *w; UAS-dpp* | 3 | Georg Halder |
| *w; UAS-Dl; UAS-tkv^act^* | 3 | This study |
| *W; UAS-tkv^act^. UAS-hh* | 3 | This study |
| *w; UAS-eya, UAS-so* | 3 | Pappu, et. al., 2003 |
| *w; UAS-eya* | 3 | Pignoni, et.al.,1997 |
| *w; UAS-so* | 3 | Pignoni, et.al.,1997 |
| *w; UAS-dac7c4* | 2 | Mardon |
| *w; UAS-dac7c4; UAS-so* | 2;3 | Mardon |
| *w; UAS-soRNAi (transformant 104386)* | 2 | VDRC (Dietzl et. al., 2007) |
| *w; UAS-eyaRNAi (transformant 108071)* | 2 | VDRC (Dietzl et. al., 2007) |
| *W; UAS-sbpRNAi (transformant 105545)* | 2 | VDRC (Dietzl et. al., 2007) |
| *w; UAS-dGFP* | 3(68A) | This study |
| *w; ey3.6dGFP* | 3(68A) | This study |
| *w; ey3.6mut* | 3(68A) | This study |
| *w; FRT42D so3/CyO ; ey3.6dGFP/TM6B* | 2;3 | This study |
| *w; FRT42D so3/CyO ; ey3.6mut/TM6B* | 2;3 | This study |
| *w; eya^cliIId^ FRT40A/ CyO; ey3.6dGFP/TM6B* | 2;3 | This study |
| *w; dac^3^ FRT40A; ey3.6dGFP/TM6B* | 2;3 | This study |
| *w; dac^3^ FRT40A; ey3.6mut/TM6B* | 2;3 | This study |
| *w; dac^3^ FRT40A; UAS-eya, UAS-so* | 2;3 | This study |
| *w; smo^3^, mad^1-2^ FRT40A/CyO; UAS-eya, UAS-so* | 2;3 | This study |
| *lz-Gal4* | X | Crew, et.al., 1997 |
| *GMR-Gal4* | 2 | Freeman, et.al., 1996 |
|  |  |  |
|  |  |  |
