# Supplementary material for: Dynamic Rewiring of the Drosophila Retinal Determination Network Switches Its Function from Selector to Differentiation
Source: PLoS Genet. 2013 Aug 29;9(8):e1003731. doi: 10.1371/journal.pgen.1003731 (PMC3757064; doi:10.1371/journal.pgen.1003731)
Supplement: Table S2 — Antibodies used in this study. Antigen, host, dilution and source are indicated. (DOCX) [file pgen.1003731.s009.docx]

**Supplemental Table 2.** **Antibodies used in this study.**

| **Antigen** | **Host** | **Dilution** | **Source** |
| --- | --- | --- | --- |
| Eyes absent | Mouse | 1:100 | DSHB |
| Dachshund | Mouse | 1:250 | DSHB |
| β-Galactosidase | Mouse | 1:1000 | Promega |
| Groucho | Mouse | 1:10 | DSHB |
| ELAV | Rat | 1:500 | DSHB |
| Cubitus interruptus | Rat | 1:250 | DSHB |
| Eyeless | Rabbit | 1:2500 | Uwe Walldorf |
| GFP | Rabbit | 1:1000 | Molecular probes |
| GFP | Chicken | 1:1000 | Abcam |
| Senseless | Guinea Pig | 1:2000 | Hugo Bellen |
| Sine oculis | Guinea Pig | 1:2000 | Ilaria Rebay |
| Rabbit IgG, Alexa 488 conjugated | Goat | 1:600 | Molecular Probes |
| Rabbit IgG, Cy3 conjugated | Goat | 1:600 | Jackson ImmunoResearch |
| Rabbit IgG, Cy5 conjugated | Goat | 1:600 | Jackson ImmunoResearch |
| Mouse IgG, Alexa 488 conjugated | Goat | 1:600 | Molecular Probes |
| Mouse IgG, Cy3 conjugated | Goat | 1:600 | Jackson ImmunoResearch |
| Chicken IgG, Alexa 488 conjugated | Goat | 1:600 | Jackson ImmunoResearch |
| Rat IgG, Alexa 488 conjugated | Goat | 1:600 | Molecular Probes |
| Rat IgG, Cy3 conjugated | Goat | 1:600 | Jackson ImmunoResearch |
| Rat IgG, Cy5 conjugated | Goat | 1:600 | Jackson ImmunoResearch |
| Guinea Pig IgG, Alexa 488 conjugated | Goat | 1:600 | Molecular Probes |
| Guinea Pig IgG, Cy3 conjugated | Goat | 1:600 | Jackson ImmunoResearch |
| Guinea Pig, Cy5 conjugated | Goat | 1:600 | Jackson ImmunoResearch |
